# A Novel Peptidoglycan Binding Protein Crucial for PBP1A-Mediated Cell Wall Biogenesis in *Vibrio cholerae*

**DOI:** 10.1371/journal.pgen.1004433

**Published:** 2014-06-19

**Authors:** Tobias Dörr, Hubert Lam, Laura Alvarez, Felipe Cava, Brigid M. Davis, Matthew K. Waldor

**Affiliations:** 1 Division of Infectious Diseases, Brigham & Women's Hospital and Department of Microbiology and Immunobiology, Harvard Medical School, Boston, Massachusetts, United States of America; 2 Howard Hughes Medical Institute, Chevy Chase, Maryland, United States of America; 3 Laboratory for Molecular Infection Medicine Sweden, Department of Molecular Biology, Umeå University, Umeå, Sweden; Indiana University, United States of America

## Abstract

The bacterial cell wall, which is comprised of a mesh of polysaccharide strands crosslinked via peptide bridges (peptidoglycan, PG), is critical for maintenance of cell shape and survival. PG assembly is mediated by a variety of Penicillin Binding Proteins (PBP) whose fundamental activities have been characterized in great detail; however, there is limited knowledge of the factors that modulate their activities in different environments or growth phases. In *Vibrio cholerae*, the cause of cholera, PG synthesis during the transition into stationary phase is primarily mediated by the bifunctional enzyme PBP1A. Here, we screened an ordered *V. cholerae* transposon library for mutants that are sensitive to growth inhibition by non-canonical D-amino acids (DAA), which prevent growth and maintenance of cell shape in PBP1A-deficient *V. cholerae*. In addition to PBP1A and its lipoprotein activator LpoA, we found that CsiV, a small periplasmic protein with no previously described function, is essential for growth in the presence of DAA. Deletion of *csiV*, like deletion of *lpoA* or the PBP1A–encoding gene *mrcA*, causes cells to lose their rod shape in the presence of DAA or the beta-lactam antibiotic cefsulodin, and all three mutations are synthetically lethal with deletion of *mrcB*, which encodes PBP1B, *V. cholerae's* second key bifunctional PBP. CsiV interacts with LpoA and PG but apparently not with PBP1A, supporting the hypothesis that CsiV promotes LpoA's role as an activator of PBP1A, and thereby modulates *V. cholerae* PG biogenesis. Finally, the requirement for CsiV in PBP1A-mediated growth of *V. cholerae* can be overcome either by augmenting PG synthesis or by reducing PG degradation, thereby highlighting the importance of balancing these two processes for bacterial survival.

## Author Summary

Bacteria surround themselves with a mesh-like peptidoglycan (PG) cell wall, which is essential for maintenance of cell shape and survival. While the enzymes that catalyze the assembly of the cell wall (aka penicillin-binding proteins (PBPs)) have been extensively characterized, our understanding of the factors that modulate the activities of these enzymes is less developed. Here, using a genetic screen, we identified a gene of unknown function that plays a crucial role in PBP1A-mediated cell wall synthesis in *Vibrio cholerae*, the bacterium causing cholera. *V. cholerae* mutants lacking this gene, whose protein product was re-named CsiV (for Cell shape integrity Vibrio), share many phenotypes with PBP1A mutants, including becoming spherical during stationary phase. We show that CsiV interacts with LpoA, a lipoprotein activator of PBP1A, as well as with PG. CsiV, LpoA, or PBP1A are all required for survival of *V. cholerae* lacking PBP1B, and mutants lacking any of these factors show marked changes in PG content in stationary phase. Collectively, our data suggest that CsiV acts through LpoA to promote PG biogenesis in *V. cholerae* and other vibrio species as well as in the other genera where this protein is found.

## Introduction

The bacterial cell wall is a remarkably sturdy, web-like structure composed mainly of peptidoglycan (PG), a polysaccharide mesh whose approximately parallel strands are crosslinked via peptide sidechains [1]–[3]. It forms a relatively thin layer between the inner and outer membranes of gram-negative bacteria, and a thicker layer in gram-positive bacteria, for which it is often the outermost bacterial structure. PG serves as a bacterial exoskeleton and promotes maintenance of the shape and size of bacterial cells [4]–[6]. The presence of PG allows bacteria to remain viable in environments where the osmolarity of the extracellular millieu is markedly lower than intracellular turgor pressure. Owing to PG's importance for bacterial survival, PG synthesis pathways are the target of some of our most commonly used antibiotics, including the beta lactams, cephalosporins and glycopeptides [7].

Most analyses of gram-negative cell wall biogenesis have been performed in *Escherichia coli*. In this model organism, the first extracytoplasmic step of PG assembly is polymerization of disaccharide-pentapeptide precursors ([*N*-acetylglucosamine – *N*-acetylmuramic acid]- pentapeptide) into glycan strands (transglycosylation (TG)) [8]. Subsequently, the peptide residues of these new strands are crosslinked (transpeptidation (TP)) to the existing PG, enabling expansion of the PG mesh. These TG and TP reactions are mediated by inner membrane-bound Penicillin Binding Proteins (PBPs) [9], of which two (PBP1A and PBP1B) are bifunctional, i.e. are able to catalyze both, TG and TP reactions. PBP1A and PBP1B are largely functionally redundant and conditionally essential, i.e., in the absence of one, the other becomes strictly required for growth [10], [11].

Recently, it was discovered that *in vivo*, the PG synthetic activities of *E. coli* PBP1A and PBP1B are dependent upon cognate outer membrane lipoproteins (LpoA and LpoB, respectively) [12], [13]. Some analyses suggest that both Lpo proteins activate the TP activity of their partners; however, it has also been suggested that LpoB can promote glycan chain polymerization [14]. In either case, the Lpo proteins are thought to play an essential regulatory role in PG synthesis, rather than a catalytic role. Despite the distinct localization patterns of PBP1s (inner membrane) and Lpo proteins (outer membrane), the PBP1/Lpo pairs were found to interact directly. It has been hypothesized that the activator proteins permit detection of gaps in the PG mesh, and thereby induce synthesis of new material where needed (i.e. where the cell wall has thinned, allowing for interaction between Lpos and cognate PBP1s) [8].

Enzymes that cleave PG also play an essential role in the survival and growth of gram-negative bacteria, and they are required for PG synthesis *in vivo*. Endopeptidases, which cleave the peptide bridges that link parallel glycan strands and thereby reverse the process of transpeptidation, are thought to create space into which new glycan strands can be inserted [15]–[17]. In *E. coli*, multiple conditionally essential enzymes mediate this process. One of 3 murein hydrolases must be present in order for incorporation of new material into the cell wall to occur, and cells lyse in the absence of all three [17].

*V. cholerae*, the gram-negative causative agent of the diarrheal disease cholera, contains a similar repertoire of PG synthetic enzymes as *E. coli*, including homologues of the PBP activators LpoA and LpoB [18]. As seen with the *E. coli* enzymes, *V. cholerae* PBP1A and PBP1B and their lipoprotein activators are conditionally essential. However, we have observed that *V. cholerae* lacking PBP1A or LpoA are more sensitive to a variety of stressors than are wt bacteria or those lacking PBP1B/LpoB, suggesting that PBP1A plays the dominant role in *V. cholerae* PG synthesis [18]. PBP1A-deficient cells appear to be particularly impaired in stationary phase, during which they lose their typical rod shape and adopt a spherical morphology. *V. cholerae* also produces functionally redundant endopeptidases that are required for cell elongation and survival, although their absence does not result in bacterial lysis [16]. Thus, *V. cholerae* and *E. coli* appear to rely on similar but not identical processes for cell wall synthesis, expansion, and maintenance.

One notable difference between *V. cholerae* and *E. coli* PG results from *V. cholerae's* production of non-canonical D-amino acids (DAA), i.e., DAA other than D-Ala and D-Glu, which are typical components of PG peptide side chains [2], [19]. As *V. cholerae* enters stationary phase, its periplasmic amino acid racemase BsrV enables it to produce additional DAA, predominantly D-Met and D-Leu, which are incorporated into PG [20], [21]. Mutants unable to produce or incorporate non-canonical DAA into PG are hypersensitive to osmotic stress, suggesting that the strength of stationary phase PG is modulated by these DAA. Interestingly, DAA likely contribute to the altered shape and survival of stationary phase *V. cholerae* lacking PBP1A or LpoA, as these mutants cease growth and assume a spherical shape in the presence of ∼1 mM concentrations of D-Met. In contrast, the growth and morphology of wild type and PBP1B/LpoB-deficient cells is unperturbed by exposure to DAA. However, the precise role of DAA in stationary phase, and the means by which they modulate *V. cholerae* PG, remain to be identified.

Here, with the aim of increasing our understanding of the processes modulated by DAA, we screened an ordered transposon library of *V. cholerae* for additional mutants that are sensitive to growth inhibition by DAA. Besides the expected insertions in the genes encoding PBP1A and LpoA, the screen was answered by an insertion in a gene of unknown function (*vc1887*), which we have subsequently renamed CsiV (for cell shape integrity Vibrio). A mutant lacking CsiV shares numerous additional phenotypes with mutants lacking PBP1A or LpoA, although the three mutants are not identical in all assays. In particular, only the effect of *csiV* disruption could be moderated by deletion of *shyA*, which encodes an endopeptidase that hydrolyzes peptide crosslinks between PG strands. Biochemical analyses revealed that CsiV interacts both with PG and with LpoA. Collectively, our data suggests that PBP1A-mediated PG synthesis in *V. cholerae* is largely dependent upon the presence of CsiV, which likely modulates the activity of the PBP1A activator LpoA.

## Results

### Identification of potential PBP1A pathway mutants by a chemical synthetic lethal screen

**Figure 1:**
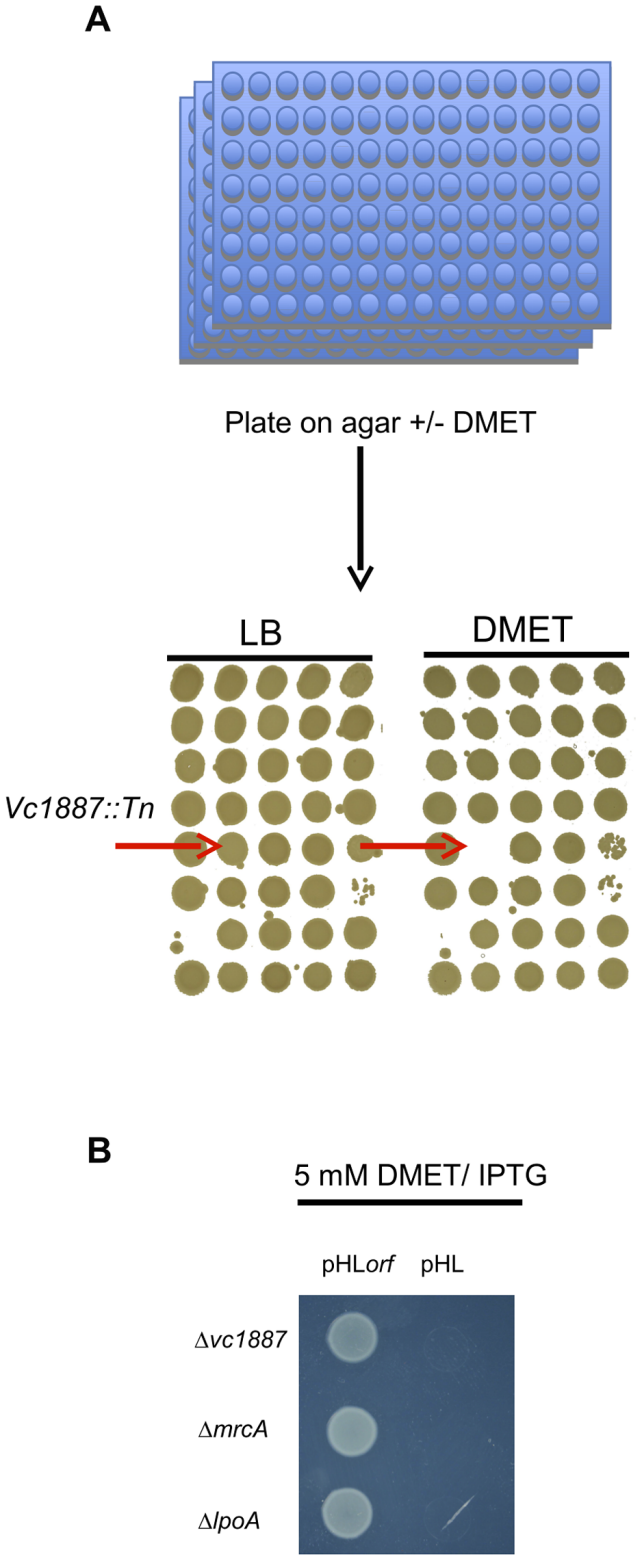
Identification and complementation of PBP1A pathway mutants using a chemical genetic screen. (A) For the screen, an ordered *V. cholerae* transposon library was pintooled onto LB agar +/−5 mM D-Met and scored for growth deficiency in the presence of D-Met. (B) Growth of *V. cholerae* PBP1A pathway mutants containing either an empty vector (pHL) or a plasmid enabling inducible expression of the deleted gene (pHL*orf*) on plates containing inducer and 5 mM D-Met confirms that their growth deficiencies on D-Met are due to deletion of the indicated genes.

We analyzed the growth of an arrayed *V. cholerae* transposon library on agar containing 5 mM D-Methionine (D-Met) (**Fig. 1A**). Of the 3,156 mutants in the library, only three were unable to grow under these conditions: strains with transposon insertions in *mrcA* (which encodes PBP1A), in *lpoA* (*vc0581*; which encodes a putative PBP1A activator [18]), and in *vc1887*, whose putative product is annotated as a hypothetical protein. In-frame deletions of *vc1887* and *lpoA* also prevented growth of *V. cholerae* in the presence of D-Met, as reported for deletion of *mrcA* (**Fig. 1B**; [19]). Furthermore, growth of each mutant could be restored by ectopic expression of the deleted gene, thereby demonstrating that the mutations do not have polar effects and that the observed growth deficiency is due to the absence of the deleted genes (**Fig. 1B**). Collectively, these results indicate that PBP1A, LpoA, and VC1887 are all required for survival of *V. cholerae* in the presence of DAA, and raise the possibility that VC1887, like LpoA, makes a key contribution to PBP1A-mediated PG synthesis. Based on our subsequent analyses of VC1887 (detailed below), we have renamed VC1887 as CsiV (for **c**ell-**s**hape **i**ntegrity in ***V*** *ibrio*).

**Figure 2:**
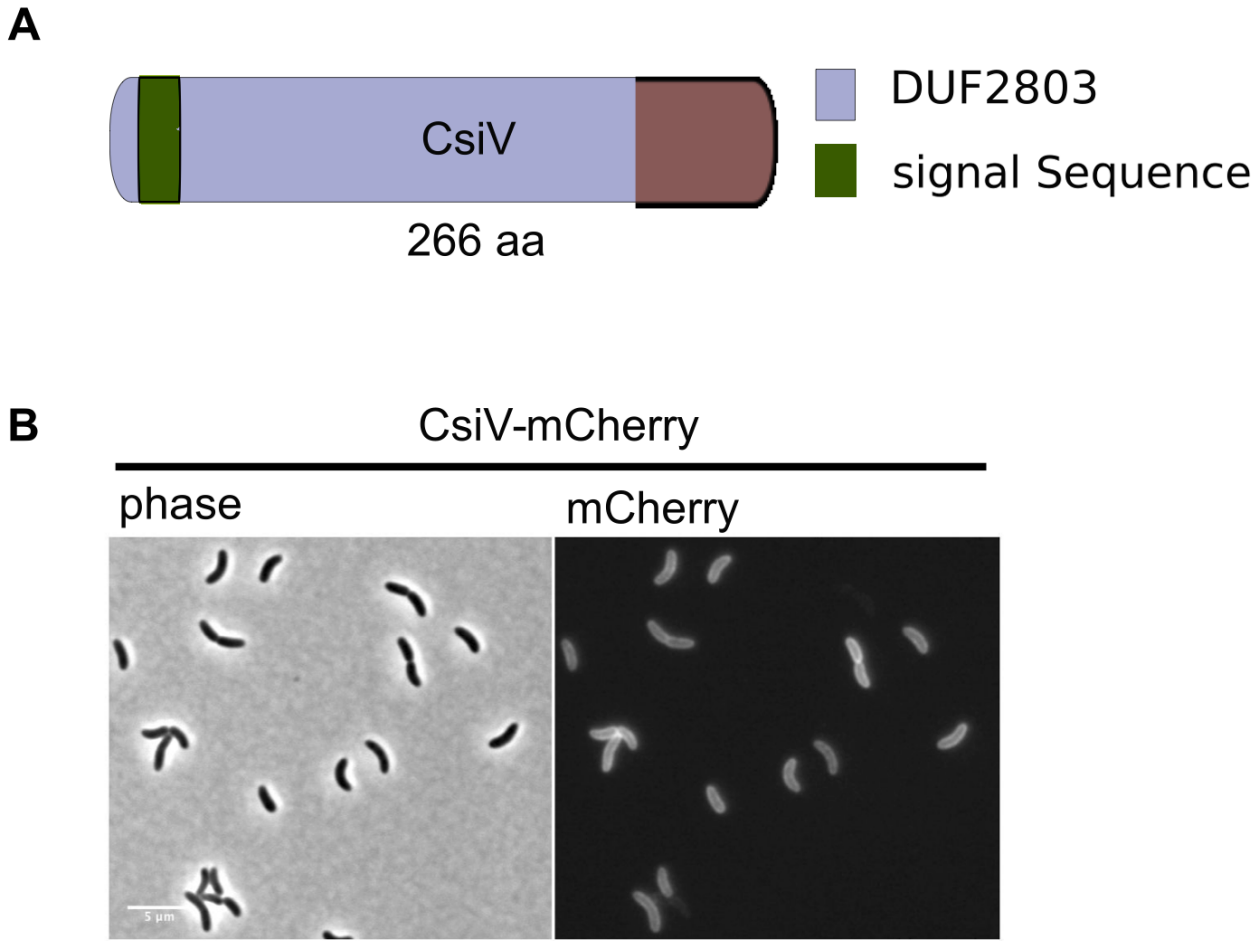
CsiV is a periplasmic protein of unknown function. (A) Domain architecture of CsiV (VC1887). The signal sequence is predicted to be cleaved off between A30 and R31. DUF2803 is not associated with any function. (B) Fluorescence microscopy analysis of *V. cholerae* producing plasmid-encoded CsiV-mCherry, a functional fusion protein that localizes to the cell periphery. Scale bar = 5 µm.

The amino terminus of CsiV is predicted to encode a signal sequence for export to the periplasm (**Fig. 2A**), and, consistent with this prediction, a CsiV-mCherry-fusion (**Fig. S1**) was targeted to the cell periphery, where it was diffusely distributed (**Fig. 2B**). Based on a String Database search for CsiV homologues, CsiV contains no additional domains with a known function in any bacterial genome. CsiV is largely restricted to *Vibrionaceae* and certain Alteromonadales (especially genus *Shewanella*) as well as *Pseudomonas sp*, with the strongest homologues only present within the genus *Vibrio* (**Fig. S2**). Structural prediction analysis (Phyre2; http://www.sbg.bio.ic.ac.uk/phyre2/html/page.cgi?id=index) did not identify high confidence structural homologues for any portion of the CsiV protein sequence, and the majority of the protein was predicted to be disordered. Thus, sequence analysis did not provide any clues regarding CsiV's function.

### A Δ*csiV* mutant is phenotypically similar to PBP1A pathway mutants

**Figure 3:**
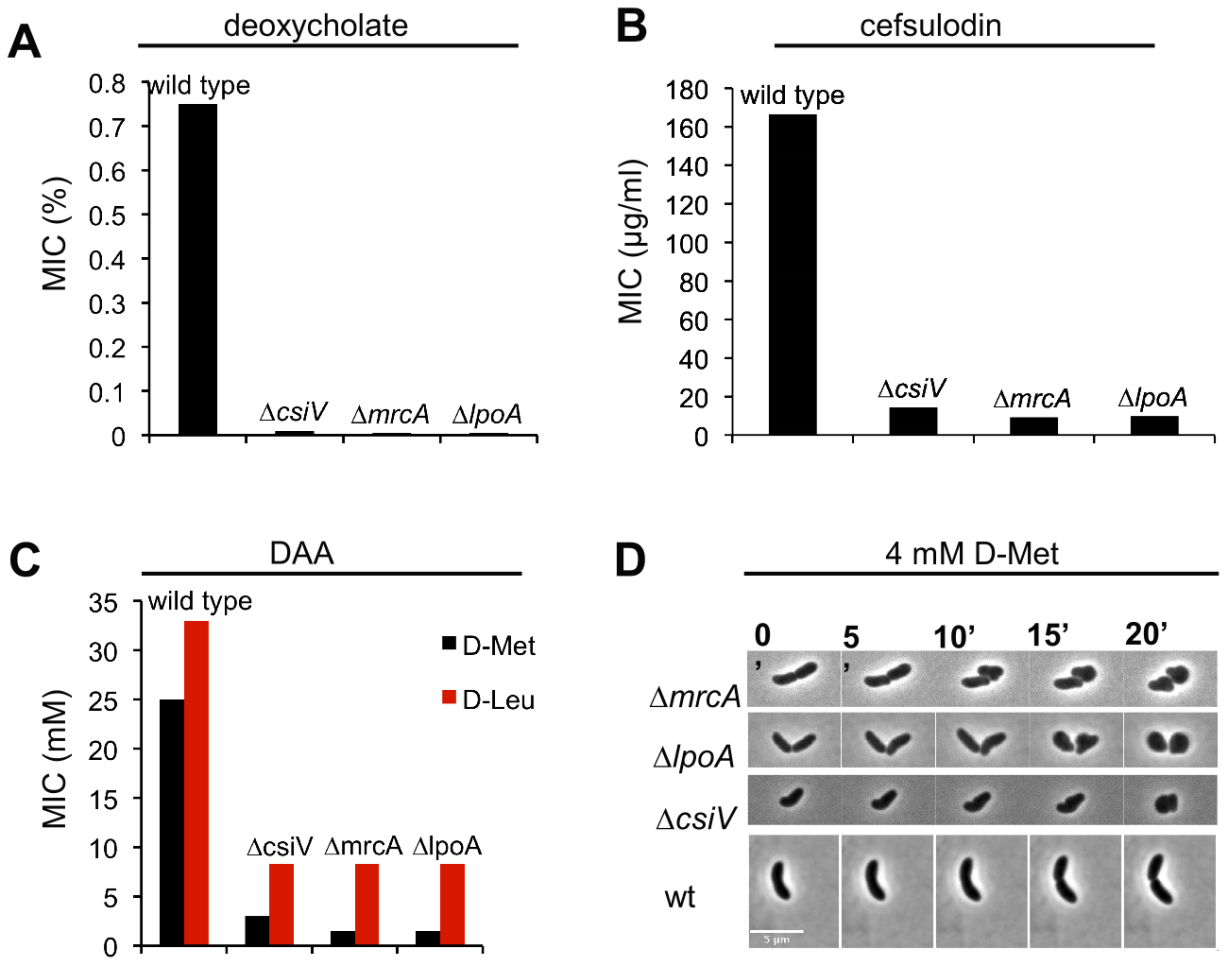
A *csiV* mutant phenocopies the growth deficiencies of *mrcA* and *lpoA* mutants. The minimum inhibitory concentration (MIC) of (A) deoxycholate, (B) cefsulodin and (C) DAA are shown for wt *V. cholerae* and PBP1A pathway mutants. MIC data reflect values obtained with two biological replicates done in technical quadruplicates for each strain. Absence of error reflects that the same values were obtained for all experiments (D) Morphological changes induced by DAA. The indicated *V. cholerae* strains were grown in the absence of DAA until OD_600_∼0.3 and then applied to 0.8% agarose pads containing 4 mM D-Met. Scale bar = 5 µm.

Given the similar responses of the Δ*csiV*, Δ*mrcA*, and Δ*lpoA* mutants to D-Met, we tested whether the Δ*csiV* strain shared other characteristics with PBP1A pathway mutants, such as loss of rod shape after exposure to DAA, as well as sensitivity to detergents and beta-lactam antibiotics [19]. These analyses revealed numerous attributes that are shared among all 3 strains. The minimum inhibitory concentrations (MIC) of deoxycholate and cefsulodin were dramatically reduced (>100 fold and ∼10 fold, respectively) for all three strains compared with wild type *V. cholerae* (**Fig. 3A,B**), and the MIC for DAA was reduced 5–10 fold (**Fig. 3C**). Furthermore, all three strains turned spherical in the presence of D-Met (**Fig. 3D**), and the process of sphere formation was comparable for the Δ*csiV*, Δ*mrcA*, and Δ*lpoA* strains (**Fig. 3D**). At first, small blebs were evident protruding from the cylindrical portion of the cell (the site of cell elongation); then, within the subsequent ∼5–10 min, DAA induced a catastrophic loss of cell shape. Thus, the three mutants appear to display similar sensitivities to a range of stresses thought to target the cell envelope. Finally, perhaps consistent with increased susceptibility to cell envelope stresses, Δ*csiV* was also similarly defective in colonization of infant mice as the PBP1A pathway mutants (**Fig. S3**).

### Comparative analyses of PG from wt and DAA-susceptible strains

**Figure 4:**
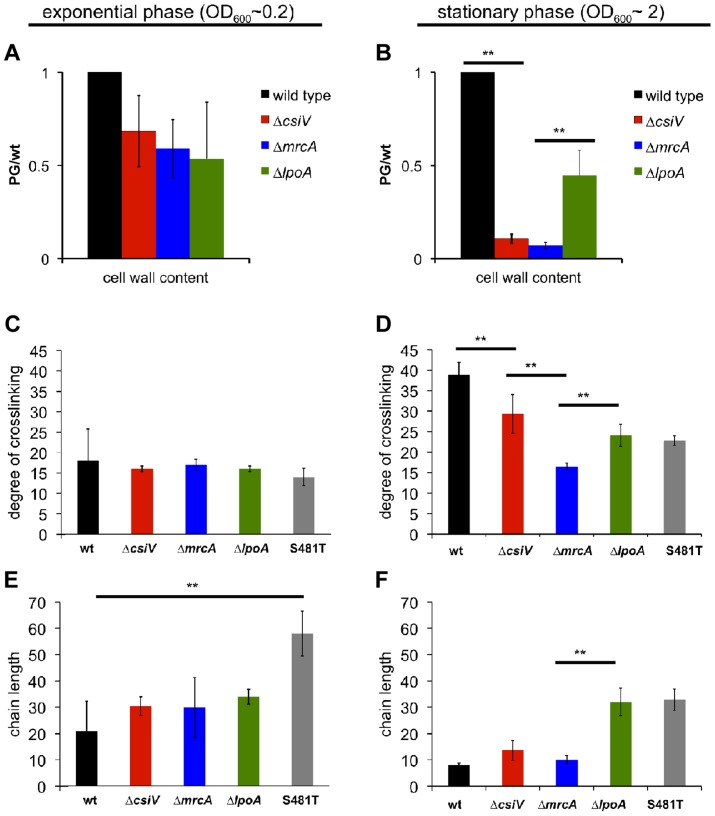
Muropeptide analysis of the *csiV*, *mrcA* and *lpoA* mutants. Cell wall content was estimated by colorimetric quantification of DAP in PG extracted from (A) exponential phase or (B) stationary phase cultures. (C–F) UPLC analysis of the degree of crosslinking (C,D) and average chain length (E, F) in cell wall extracted from exponential phase (C,E) and stationary phase (D,F) cultures. Data are averages of two (exponential phase) or three (stationary phase) biological replicates, error bars indicate standard deviation. ** = p<0.01 (t-test). S481T is a PBP1A transpeptidase mutant expressed from its native chromosomal locus, where the S residue at position 481 (in the active site) is replaced by a T.

Given PBP1A's prominent role in PG synthesis, we also compared PG content and composition for wt *V. cholerae* and the three mutants. Based on the abundance of diaminopimelic acid (a PG constituent) in cell wall material isolated from exponential phase cultures, 50–90% of wild type PG (normalized to OD_600_) could be recovered from all three mutants, suggesting cells can compensate almost fully for the loss of the PBP1A pathway and CsiV during exponential phase. In contrast, for stationary phase cultures, PG recovery from both the Δ*mrcA* and Δ*csiV* mutants was ∼90% less than from wild type *V. cholerae* (**Fig. 4A**). The Δ*lpoA* mutant also contained markedly less PG than the wild type strain, albeit ∼5 fold more than the Δ*mrcA* and Δ*csiV* mutants. These results suggest that PBP1A is responsible for a high proportion of *V. cholerae* PG synthesis during the transition into or in stationary phase, and suggest that this synthesis may be dependent upon CsiV.

To gain insight into the regulation of PBP1A's transpeptidase (TP) and transglycosylase (TG) activities and their connection to CsiV, we also compared the degree of crosslinking and the length of glycan chains in PG from the wt and mutant strains. For these analyses, we included a *mrcA* mutant (S481T) predicted to produce PBP1A that lacks TP activity, due to disruption of the enzymatic active site. We found that crosslinking was slightly and insignificantly reduced, relative to the wt strain, in exponential phase-derived PG from all four mutants (Δ*csiV*, Δ*mrcA*, Δ*lpoA*, *mrcAS481T*) (**Fig. 4C**). In contrast, crosslinking was significantly reduced in stationary phase PG in all four mutants (**Fig. 4D**), although deletion of *csiV* resulted in less reduction than did deletion of *mrcA*. These results, consistent with our analysis of PG content, suggest that while PBP1A is largely dispensable in exponential phase, it is linked to a significant fraction of PG crosslinking in stationary phase, and that CsiV may promote (but not be required for) PBP1A's TP activity.

Unexpectedly, the average PG chain length (number of GlcNac-MurNac subunits/chain) differed markedly between mutants and growth phases (**Fig. 4E,F**). While all four mutants had slightly longer PG chains (though this difference was only significant for the *mrcA*S481T mutant) than the wild type in exponential phase (**Fig. 4E**), the Δ*lpoA* and S481T mutants had significantly longer glycan chains in stationary phase (**Fig. 4F**), where in contrast chain length of Δ*csiV* and Δ*mrcA* did not differ from the wild type. The similarity between the Δ*lpoA* and *mrcAS481T* strain in this assay suggests that LpoA may be particularly important for augmenting PBP1A's TP activity (as has also been observed in *E. coli*) during the transition into or in stationary phase. However, our results also suggest a possible interplay between PBP1A's two enzymatic activities, such that disruption of its TP activity may cause deregulation of its TG activity. Additionally, it is noteworthy that in our analyses of PG, unlike previously described assays, the absence of the putative non-enzymatic factors (LpoA and CsiV) does not always have the same consequences as the absence of PBP1A.

### CsiV shares genetic interactions with PBP1A and LpoA

**Figure 5:**
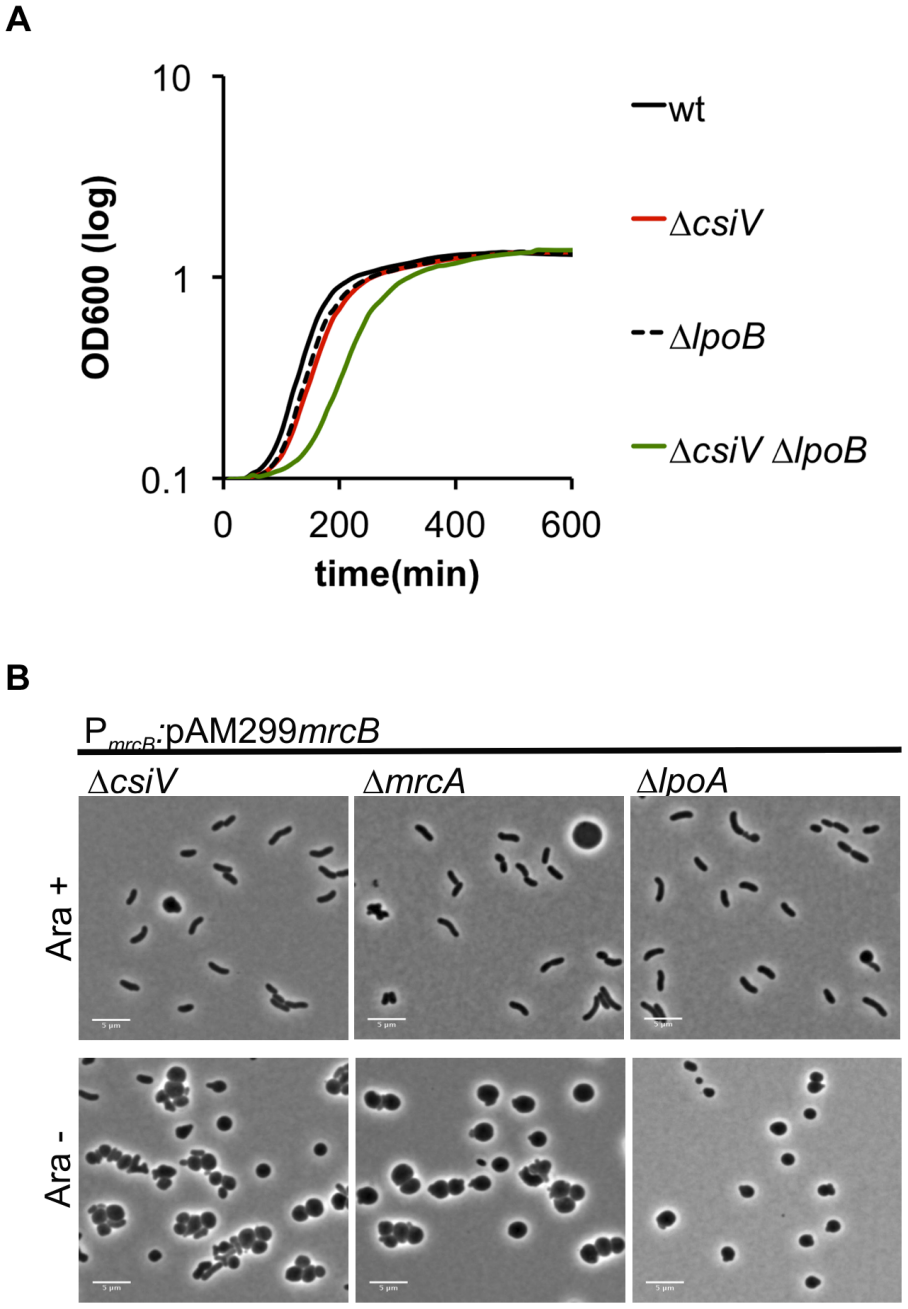
Deletion of *csiV* is synthetically lethal with deletion of *mrcB* but not *lpoB*. (A) Growth curves, based on OD_600_, for the indicated *V. cholerae* strains grown in LB. Data represent averages of technical triplicates and are representative of two experiments with similar results. (B) Cells in which PBP1B's native promoter was replaced by an arabinose-inducible promoter (P_ARA_) were initially grown in the presence of arabinose (Ara^+^), then grown for 1 h in fresh medium without arabinose (Ara^−^).

In previous work, we have shown that mutations in *V. cholerae mrcA* and *lpoA* are synthetically lethal with mutations in *mrcB* and *lpoB* [18], i.e., that the PBP1B pathway is essential in the absence of either PBP1A or LpoA. Similarly, we were unable to generate an in-frame deletion of *mrcB* in the Δ*csiV* background, strongly suggesting that PBP1B is also essential in this mutant, possibly because PBP1A activity in a Δ*csiV* Δ*mrcB* mutant is insufficient to sustain growth. Unexpectedly, we were able to generate a Δ*csiV* Δ*lpoB* mutant, although this strain did have a modest growth deficiency (e.g., a longer lag phase) (**Fig. 5A**). Presumably, either PBP1A or PBP1B (or perhaps both) has reduced activity, rather than a total loss of function, in this double mutant. This possibility is explored further below.

By placing *mrcB* under the control of an arabinose-inducible promoter in the wt, Δ*mrcA*, Δ*lpoA* and Δ*csiV* backgrounds, we were able to observe the consequences of PBP1B depletion in various genetic backgrounds. When grown in the presence of arabinose, the majority of cells displayed normal cell morphology in all 4 strains (**Fig. 5B**, **S4** and not shown). However, when arabinose was removed (resulting in PBP1B depletion), almost all of the Δ*mrcA*, Δ*lpoA* and Δ*csiV* cells became spherical, as observed following exposure of these strains to DAA. In contrast, PBP1B depletion had no effect on the morphology of otherwise wt cells (data not shown). The similarity between changes in the mutants' cell shape in response to DAA and to the absence of PBP1B suggests that a key effect of DAA in *V. cholerae* may be to inhibit PBP1B.

### PBP1A function is not entirely dependent upon CsiV

**Figure 6:**
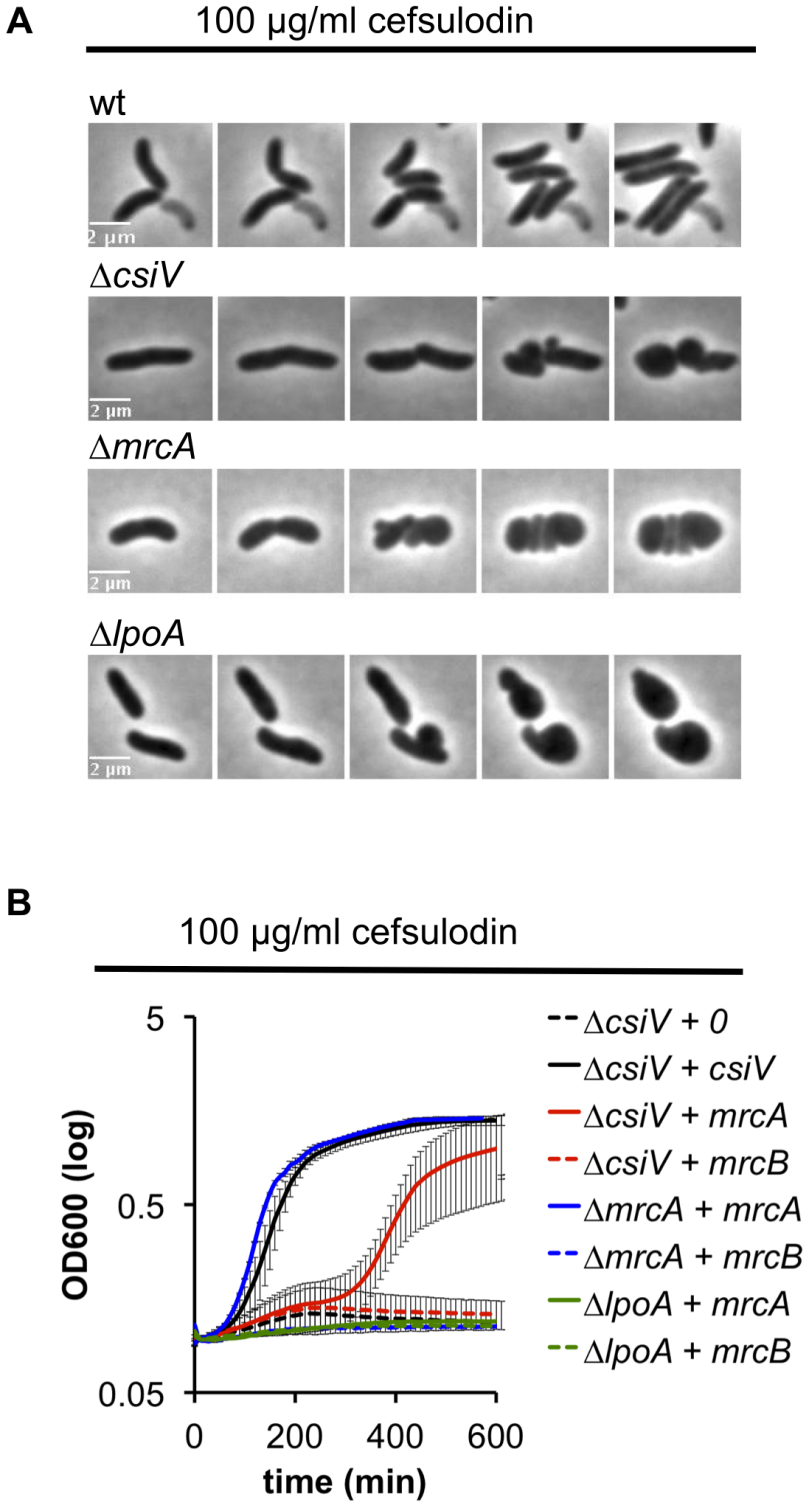
*V. cholerae* PBP1A is partially functional in the absence of CsiV. (A) Morphological changes of *V. cholerae* strains grown in the presence of the PBP1B inhibitor cefsulodin. Frames are 5 min apart. (B) Growth (OD600) of Δ*csiV*, Δ*mrcA* and *ΔlpoA* strains ectopically expressing *mrcA* or *mrcB* and cultured in the presence of cefsulodin. Data are averages of three experiments, error bars represent standard deviation.

We also explored the requirement for CsiV, LpoA, and PBP1A in PBP1B-deficient cells by treating the panel of mutant strains with cefsulodin, which specifically inhibits PBP1B in *V. cholerae* [18]. When all proteins were expressed at endogenous levels, the strains failed to grow in 100 µg/ml cefulodin, and instead adopted a spherical morphology (**Fig. 6A**), as seen in response to PBP1B depletion. However, when PBP1A was overproduced, CsiV-deficient cells were able to grow in the presence of cefsulodin (albeit not as well as when CsiV was exogenously produced) (**Fig. 6B**), providing further evidence that PBP1A is not fully inactive in the absence of CsiV. Alternatively, the observed phenotype could indicate the rise of a resistant mutant or degradation of the antibiotic under these conditions; however, these alternative explanations seem unlikely since we never observed growth in the control (Δ*csiV* carrying empty plasmid+cefsulodin). In contrast to Δ*csiV*, neither overexpression of PBP1A nor of CsiV (data not shown) enabled LpoA-deficient cells to grow in the presence of the antibiotic, indicating that, as in *E. coli*, LpoA is absolutely required for PBP1A function.

### Mutation of an elongation-specific D,D-endopeptidase prevents sphere formation in the Δ*csiV* mutant

**Figure 7:**
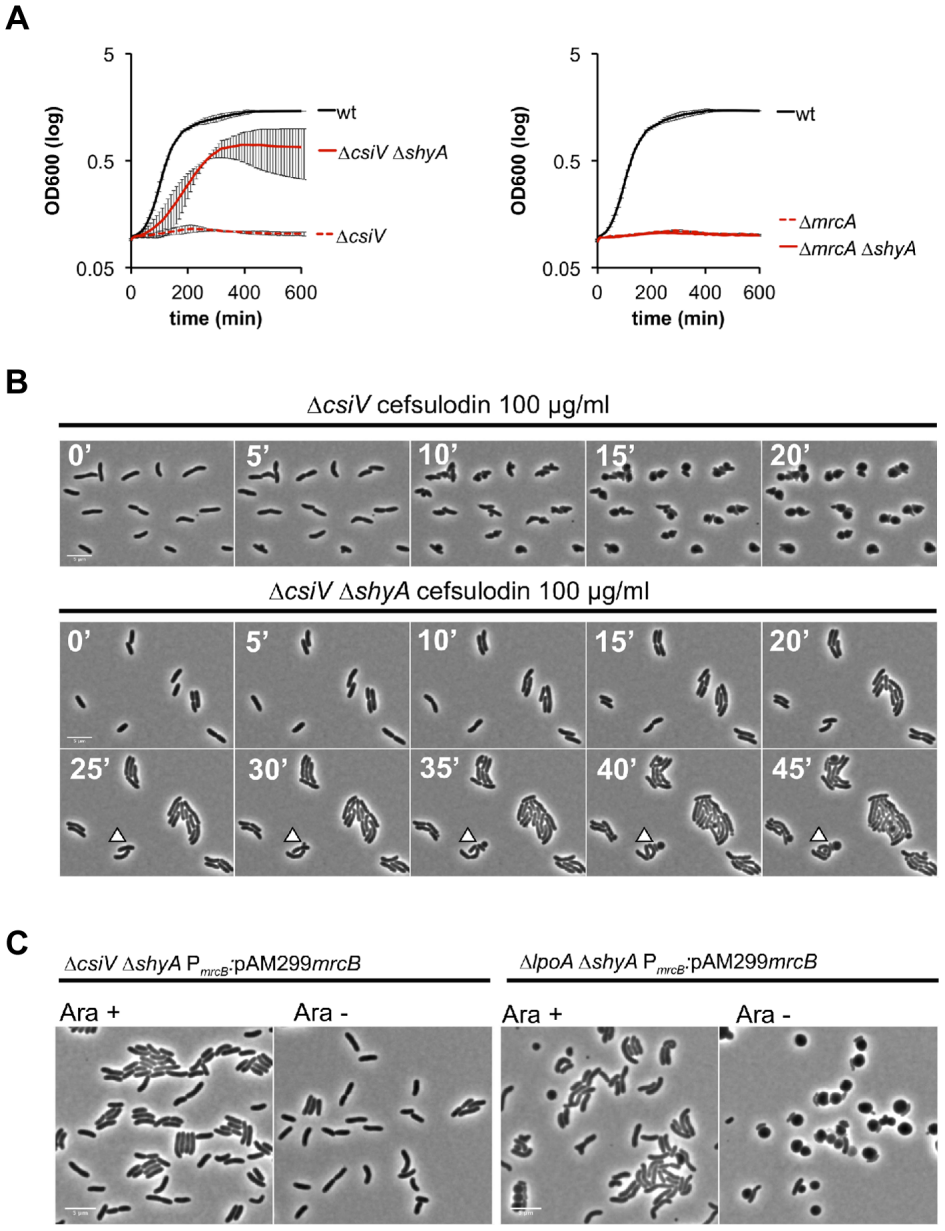
The absence of the endopeptidase ShyA enables PBP1B-independent growth of CsiV-deficient *V. cholerae*. (A) Growth curves, based on OD_600_, for cultures containing the PBP1B inhibitor cefsulodin. Data are averages of technical quadruplicates and representative of 4 experiments with similar results, error bars represent standard deviation (B) Single-cell dynamics of Δ*csiV* and the Δ*csiV ΔshyA* double mutant in the presence of cefsulodin. Cells were grown to OD_600_∼0.3 and then applied to agarose pads containing 100 µg/ml cefsulodin. Arrowheads point to blebs (C) Δ*csiV ΔshyA* or Δ*lpoA ΔshyA* cells in which PBP1B's native promoter was replaced with an arabinose-inducible promoter (P_ARA_) were treated as described in **Fig. 5B**.

To gain additional insight into the cellular role of CsiV, including its relationship to PBP1A and LpoA, we screened transposon insertion libraries generated in the Δ*csiV*, Δ*mrcA*, and Δ*lpoA* strains for mutants that had regained the ability to replicate in the presence of DAA. No suppressor mutations were obtained for the Δ*mrcA* and Δ*lpoA* mutations; however, multiple independent insertions within *shyA* (*vca0079*) were found to enable growth of the Δ*csiV* mutant in the presence of DAA. The product of *shyA*, which is one of two functionally redundant periplasmic hydrolases required for cell elongation in *V. cholerae* ([16]), can cleave the majority of peptide crosslinks in *V. cholerae* PG. Subsequent analyses revealed that an in-frame deletion in *shyA* was also necessary and sufficient to significantly mitigate the growth and morphology defects of the Δ*csiV* strain in the presence of DAA (not shown) and cefsulodin (**Fig. 7 A,B**, **S5**) and in response to PBP1B depletion (**Fig. 7C**), while deletion of *shyA* did not enable growth or maintenance of normal cell shape for PBP1A or LpoA-deficient strains under these conditions (**Fig. 7A,C**). A likely explanation for these data is that a small amount of PBP1A activity is still preserved in the Δ*csiV* mutant (but not the Δ*mrcA* or Δ*lpoA* strains), and that deletion of *shyA* reduces PG cleavage and thereby lessens the need for PG synthesis to a level that can be met by residual PBP1A activity. However, it is also possible that CsiV does not modulate PBP1A activity at all, but instead restrains the activity of ShyA, i.e., that the PG-related phenotypes of the Δ*csiV* strain are the consequences of elevated PG degradation rather than reduced synthesis. In theory, CsiV might modulate both synthetic and degradative processes.

To explore whether ShyA deregulation in the absence of CsiV might account for some of the phenotypes of the Δ*csiV* mutant, we assessed the effect of ShyA overexpression in the Δ*csiV*, Δ*mrcA*, and Δ*lpoA* strains. Notably, ectopic expression of ShyA, which we have previously shown to be functional [16], had no effect on the growth rate of the 3 mutants or of wt cells (**Fig. S6**), and all 4 strains maintained *V. cholerae's* normal rod morphology (not shown). Thus, although ShyA activity is likely to be highly regulated *in vivo* ([16]), to date we lack evidence that its activity is restrained by CsiV or components of the PBP1A pathway.

### CsiV interacts with LpoA

Analyses of CsiV interaction partners also suggests that CsiV probably modulates *V. cholerae* cell shape and growth predominantly via an effect on PG synthesis rather than degradation. We performed affinity purification analyses, using His-antibody resin and 6× His-tagged CsiV expressed from its native chromosomal location, to identify proteins that interact with CsiV (**Fig. S7A**). To stabilize protein complexes, some cells were treated with the crosslinker DSP (dithiobis succinimidyl propionate, Lomant's reagent) prior to lysis. Silver staining of column-purified proteins, followed by mass spectrometry analysis of bands of interest, revealed that LpoA copurified with CsiV even in the absence of crosslinker. Purification of an additional protein complex, which contained both LpoA and VC2168, a small, predicted periplasmic protein of unknown function, was found to depend on crosslinking. Most additional protein bands were found to contain chaperones, ribosomal proteins, or CsiV. Comparable analyses, using lysates from +/− DSP-treated cells expressing LpoA-His6, confirmed the crosslinker-independent co-purification of LpoA and CsiV and the crosslinker-dependent co-purification of LpoA and VC2168 (**Fig. S7B**). Given the stringency of washing conditions used (500 mM NaCl), our data suggest that a high affinity interaction occurs between LpoA and CsiV, consistent with CsiV modulating PBP1A-mediated PG synthesis. The additional interaction partner, VC2168, has a high degree of phylogenetic co-occurrence with CsiV (string database, **Fig. S2**), suggesting it may likewise play a role in cell envelope biogenesis. However, we have yet to identify any changes in cell growth or morphology associated with deletion of *vc2168*, and its cellular role remains obscure. Somewhat unexpectedly, our mass spectrometry-based analyses did not detect any interaction between PBP1A and either CsiV or LpoA.

**Figure 8:**
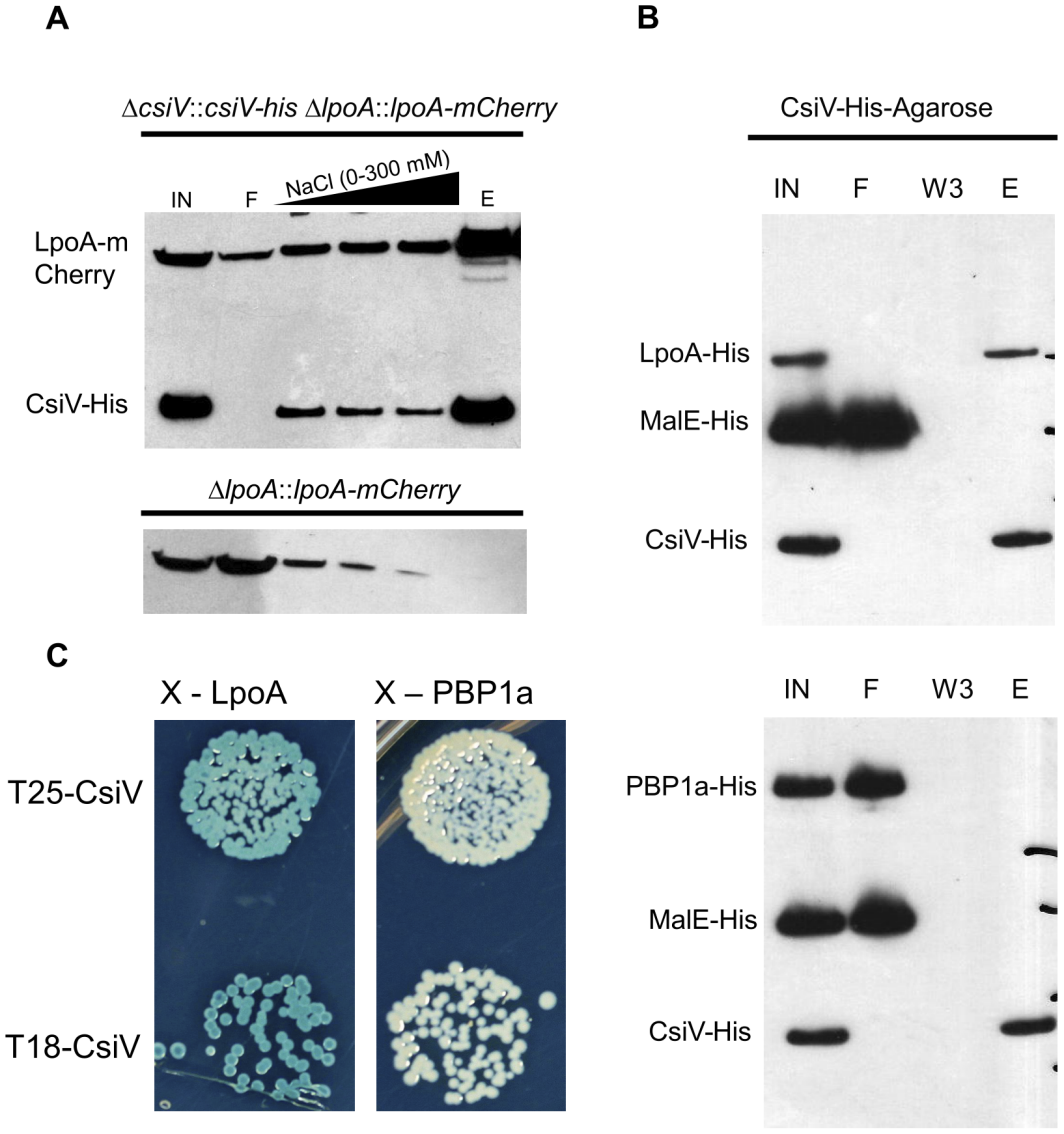
CsiV interacts with LpoA. (A) Lysates from a *csiV::csiV-his lpoA::lpoA-mCherry* strain and a Δ*lpoA::lpoA-mCherry* strain were affinity purified on NiNTA resin, then Western blotted with anti His and anti-mCherry antibodies to show copurification of CsiV and LpoA. (B) Purified MalE-His (10 µM) and either LpoA-His_77-653_ (2.5 µM) or PBP1A-His (2.5 µM) were mixed with CsiV-His_31-266_ covalently attached to NHS-activated sepharose. Protein associations were assessed via western blotting (anti-His) of Input (IN), flowthrough (F), third wash (W3), and eluate from boiled beads (E). (C) Bacterial two-hybrid analysis of CsiV-LpoA and CsiV-PBP1A interactions. All constructs are truncated downstream of their signal sequences to allow for cytoplasmic BACTH.

The interaction between CsiV and LpoA, but not PBP1A was evident in a variety of additional assays as well. Western blotting of affinity-purified proteins expressed from chromosomal loci confirmed that purification of LpoA was mediated by an interaction with CsiV-His6 (**Fig. 8A**). Furthermore, when His6-tagged purified LpoA_61-433_ or PBP1A (as well as a control protein, MalE) were loaded on a column containing truncated CsiV (CsiV_31-266_) covalently linked to NHS-activated Sepharose, only LpoA was retained by the column (**Fig. 8B**). Finally, using a split adenylate cyclase-based bacterial two-hybrid assay, CsiV was found to interact with LpoA but not PBP1A (**Fig. 8C**) or ShyA (data not shown). Thus, although all our interaction assays do not rule out the possibility of interactions between CsiV and partners other than LpoA, our observations provide strong support for the hypothesis that CsiV modulates *V. cholerae* PG synthesis, and thereby affects cell shape and growth, via directly interacting with LpoA and promoting its function as an activator of PBP1A.

### CsiV interacts with peptidoglycan

**Figure 9:**
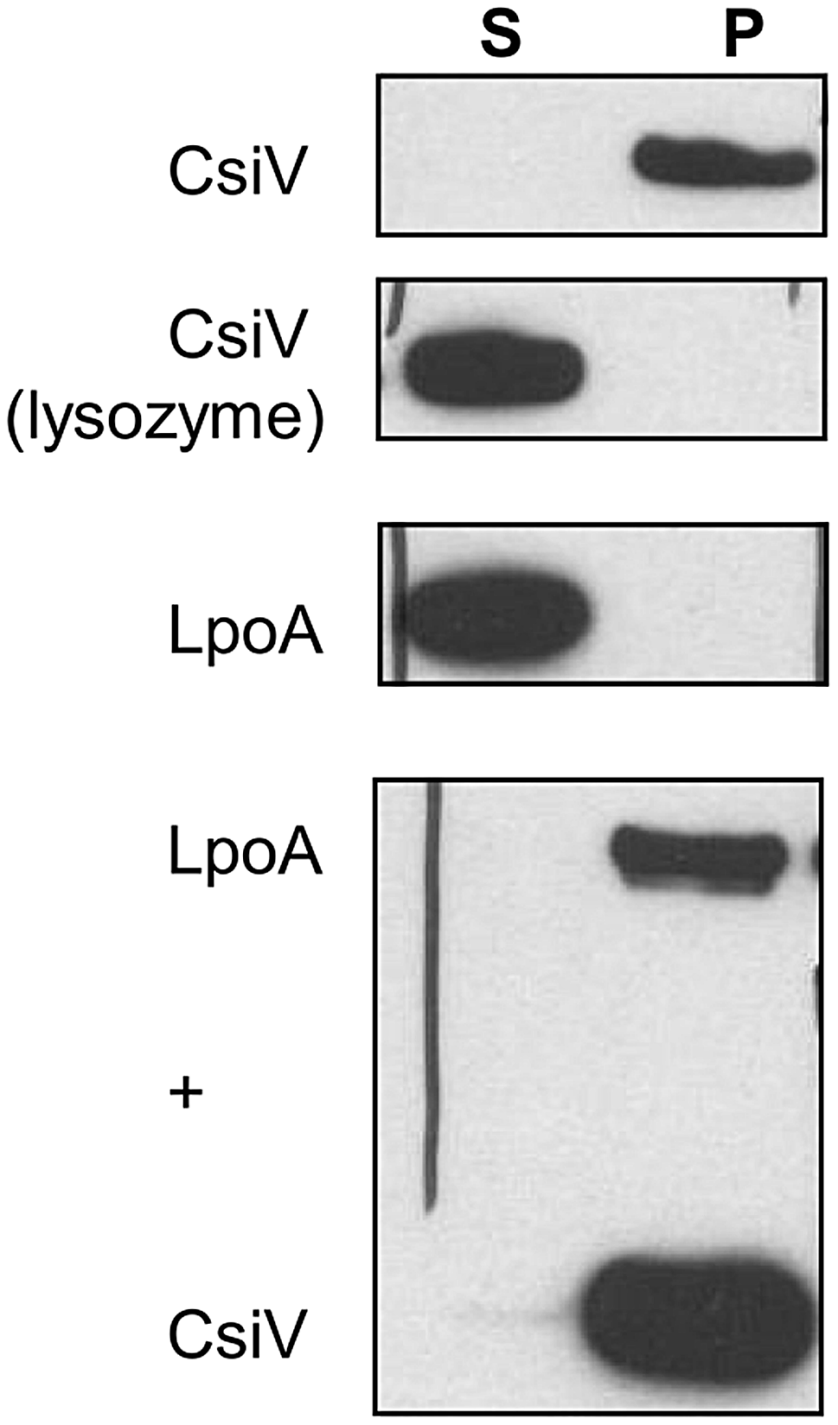
CsiV interacts with peptidoglycan. Purified proteins were incubated with SDS-extracted PG, then ultracentrifuged to pellet PG and associated proteins. Proteins were visualized by anti-His Western blot. For the lysozyme control, 1 mg/ml lysozyme was added simultaneously with the addition of CsiV-His.

Since periplasmic enzymes involved in cell wall biosynthesis are necessarily closely associated with the cell wall, we tested whether CsiV directly interacted with peptidoglycan. We incubated purified CsiV with purified PG and then pelleted PG using ultracentrifugation. CsiV exclusively associated with the pellet fraction in the presence of PG, but not when lysozyme was added to the reaction, suggesting a direct interaction between CsiV and the cell wall (**Fig. 9**). Interestingly, purified LpoA did not interact with PG by itself but was tethered to it by the simultaneous presence of CsiV. This interaction did not appear to be required for LpoA-PG interaction *in vivo*, as PG purified after treatment of cells with DSP (which can mediate covalent attachment of proteins to the cell wall as well as crosslinking of protein complexes [22]) retained natively expressed LpoA-His even in the absence of PBP1A and CsiV (**Fig. S8**).

## Discussion

Here, we discovered CsiV, a new player in cell wall biogenesis in *V. cholerae* and presumably in all vibrios as well as the other genera where strong homologues of this novel peptidoglycan-binding protein are found. CsiV plays a critical role in PBP1A-mediated cell wall biogenesis in *V. cholerae* and was identified by screening a mapped transposon library for mutants whose growth was inhibited by D-amino acids (DAA). The screen was inspired by our work that revealed that D-amino acids are key modulators of cell wall synthesis, particularly as cells enter stationary phase [19]. D-amino acids inhibit growth of strains lacking PBP1A or its putative activator LpoA, and exposure of these mutants to DAA leads to loss of rod shape and sphere formation through an unknown mechanism [18]. CsiV-deficient cells, like PBP1A and LpoA-deficient cells, turn spherical in stationary phase, in the presence of DAA, and upon depletion of PBP1B. Additionally, *V. cholerae* lacking CsiV, LpoA, or PBP1A are hypersensitive to the bile acid deoxycholate and to cefsulodin, which inhibits *V. cholerae* PBP1B, and they all show marked changes in PG content in stationary phase. CsiV, LpoA and PBP1A are also all required for survival of *V. cholerae* lacking PBP1B, although the requirement for CsiV is diminished in the absence of the endopeptidase ShyA. CsiV interacts with LpoA as well as with PG, but does not appear to bind to PBP1A. Collectively, our data suggest that CsiV acts through LpoA (and thereby through PBP1A) to promote PG synthesis in *V. cholerae*, and that *V. cholerae* PBP1A is largely inactive in the absence of CsiV.

Despite the extensive similarities of the phenotypes of *V. cholerae csiV*, *lpoA*, and *mrcA* mutants, there are also notable differences among these strains. A key difference is that overexpression of *mrcA* enables growth of the *csiV* mutant, but not the *lpoA* mutant, in the presence of cefsulodin. In the presence of this antibiotic, PBP1A-mediated PG synthesis is essential for growth; consequently, our results suggest that PBP1A retains a small amount of activity in the absence of *csiV*, but is completely inactive when LpoA is not present. Since CsiV interacts with LpoA, but does not appear to interact with PBP1A, a likely explanation for these results is that CsiV markedly enhances the ability of LpoA to activate PBP1A. Western blot analyses of the abundance of epitope tagged LpoA in the presence and absence of CsiV (**Fig. S8** and data not shown) suggest that CsiV is not required simply to stabilize LpoA levels, nor is CsiV required to tether LpoA to PG (**Fig. S8**). Our path to elucidating the precise means by which CsiV modulates LpoA's activity will become clearer as the molecular bases for LpoA's enhancement of PBP1A activity are illuminated.

In related analyses, we observed that disruption of *V. cholerae lpoB* is also possible in a *csiV* mutant, but not in an *lpoA* or *mrcA* mutant, and that disruption of *mrcB* is not possible in any of the three mutants. The former results are consistent with our previous conjecture that only the *csiV* mutant has residual PBP1A activity. Additionally, the differential requirement for *lpoB* and *mrcB* in the *csiV* mutant suggests that PBP1B may likewise have a small amount of residual activity in the absence of LpoB, and that PBP1B-mediated PG synthesis is critical for survival of the *csiV lpoB* mutant. Consistent with this supposition, we have found that growth of this strain is blocked by the PBP1B-specific antibiotic cefsulodin (data not shown).

Our studies have also revealed extensive similarities between the consequences of PBP1B depletion, exposure to cefsulodin, and exposure to DAA. All inhibit growth of PBP1A, LpoA, and CsiV-deficient cells and induce loss of rod shape and adoption of a spherical morphology. Although they do not provide conclusive evidence, these results suggest that one effect of DAA is to inhibit PBP1B activity. DAA might bind directly to PBP1B; alternatively, PG in which non-canonical DAA have been incorporated might be a poor substrate for crosslinking by PBP1B. Regardless, if effective reduction of PBP1B activity in the presence of DAA occurs, then PBP1A likely accounts for the majority of PG synthesis in stationary phase cultures, which accumulate high levels of DAA. Such a role for PBP1A would explain why the survival and PG content of PBP1A pathway mutants is particularly reduced during stationary phase.

Since the detrimental effects of *csiV* deletion in the absence of PBP1B activity were mitigated by inactivation of the endopeptidase ShyA, it is formally possible that CsiV acts as a negative regulator of PG degradation rather than an indirect activator of PG synthesis. However, this scenario seems unlikely to account for all of CsiV's activity for a variety of reasons. First, CsiV interacted with LpoA, while we did not detect an interaction between CsiV and ShyA or any other endopeptidase. Second, overexpression of ShyA did not impede *V. cholerae* growth, even in the absence of CsiV, suggesting that restraint of detrimental PG digestion is not dependent upon CsiV (**Fig. S6**). Additionally, the deletion of *shyA* promoted but did not completely restore growth of the Δ*csiV* mutant in cefsulodin, so, at minimum, the role of CsiV cannot be limited to regulation of ShyA activity. Thus, CsiV's most probable role is in promoting PG synthesis, via enhancing LpoA's activation of PBP1A.

Finally, our observations have bearing on a central question in cell wall biogenesis - whether PG synthesis and degradation are co-ordinately regulated. These key processes may be tightly coupled, so that one “degradation unit” is always associated with one “synthesis unit” of cell wall material. It is also possible that degradation and synthesis are independently regulated in response to one or more cellular stimuli. For example, degradation might occur in response to elevated turgor pressure in the cell, while synthesis might be stimulated by detection of gaps within the PG structure. In either case, our observations suggest that cell survival depends on proper maintenance of a balance between these two processes. When CsiV and PBP1B are both absent, and consequently PG synthesis rests solely on residual PBP1A activity, cell growth is dependent upon compensatory measures: either removal of the endopeptidase ShyA or overexpression of PBP1A, which should reduce PG degradation or increase synthesis, respectively. In contrast, cell growth is not markedly impaired when only a single bifunctional PBP (PBP1A or PBP1B) was absent. While speculative, our findings are therefore consistent with a scenario in which PG synthesis and degradation are buffered, rather than precisely calibrated, processes, i.e. that overall PG synthetic activity can be lowered substantially until a threshold is reached, below which degradation outweighs synthesis to a degree that makes it impossible to maintain rod-shape. This is in agreement with recent studies of the activity of PBP2, the transpeptidase essential for cell elongation, in *E. coli* [23].

## Materials and Methods

### Media and growth conditions

All strains were routinely grown at 37°C in LB medium supplemented with 200 µg/ml streptomycin. For growth curves, overnight cultures were diluted 1∶100 into fresh medium and incubated shaking until OD_600_∼0.1–0.3. Cultures were then normalized to an OD_600_ of exactly 0.1 and transferred to 200 µL volume in 200-well honeycomb plates. Growth curves were then conducted at 37°C with continuous shaking in a Biotek growth curve machine.

### Plasmid construction

Primers are summarized in Table S1.

Complementation plasmids were constructed by cloning PCR fragments amplified with primers TDP166/167 (*csiV*), TDP168/169 (*mrcA*) or TDP172/173 (*lpoA*) digested with Xma1/BamH1 (NEB) into likewise digested and Calf intestinal phosphatase (CIP,NEB)-treated pHL100.

pHL100shyA was constructed using isothermal assembly [24] of the product of primers TDP529/530 with Sma1 (NEB)-digested and CIP-treated pHL100.

pHL100csiV-mCherry was constructed using isothermal assembly of Sma1-digested/CIP-treated pHL100 and the products of TPD310/311 and TDP238/239.

All deletion plasmids are derivatives of the suicide-vector pCVD442 [25]. 300–500 bp long upstream and downstream homologies were amplified using primers **TDP138**/139+TDP140/**141** (*lpoA*), **TDP205**/206+TDP207/**208** (*mrcA*) or **TDP282**/283+TDP284/**285** (*lpoB*), purified (Qiagen PCR purification kit) and fused using SOE PCR with the respective outside primers (in bold). The resulting product was digested with Xba1 (NEB) and ligated into likewise digested pCVD442.

The csiV deletion plasmid was constructed using isothermal assembly of the products of TDP360/361+TDP362/362 into Sma1-digested pCVD442.

Overexpression plasmids for protein purification are derivatives of pET28b. Open reading frames encoding truncated CsiV and LpoA were cloned into the Nco1/Xho1 sites using the PCR products of TDP110/111 and TDP201/88. HisPBP1A was amplified using primers TDP535/536 and cloned into Nco1-digested pET28b using isothermal assembly.

### Site-directed mutagenesis

Site-directed mutagenesis was performed using the QuikChange kit (Agilent) following the manufacturer's recommendations. Primers TDP160/161 were used to amplify mutated *mrcA* from pHL100*mrcA*. Mutated *mrcA* was then amplified using primers TDP212/214 and used as template together with the products of TDP196/212+213/199 in a SOE PCR reaction with primers TDP196/199. The resulting product, containing mutated *mrcA*+upstream and downstream homology regions was then digested with Xba1 and ligated into likewise digested pCVD442.

### Strain construction

Strains and plasmids are summarized in Table S2. All *Vibrio cholerae* strains are derivatives of El Tor N16961.

Deletion and replacement mutants were generated using the suicide plasmid pCVD442 or the *lacZ* integration plasmid pJL1 [26] in the donor strain SM10 using published methodology ([25]).

### Transposon screen

An ordered transposon library in 96 well format [27] was transferred to 200 µL LB medium using a 96-Pin Tool, incubated overnight at 37°C and then spotted on LB agar plates with either no addition or 5 mM D-methionine (Sigma). After another overnight incubation, agar plates were visually inspected for growth. Colonies that grew neither on LB nor on D-Met were recultured from the library and retested for growth on D-Met individually. Colonies that grew on LB agar but not on LB were scored as hits.

### PG isolation and HPLC analysis

To isolate murein sacculi, either 1L (stationary phase culture, OD_600_∼2) or 2L (exponential phase culture, OD_600_∼0.2) of culture was pelleted, resuspended in 5 ml PBS and slowly added to 10 ml of boiling 10% SDS while stirring. Samples were boiled for 4 h, then stirred overnight at 37°C. Cell wall material was then pelleted by ultracentrifugation (110.000 rpm, 1 h) and washed 3× in MQ water.

Peptidoglycan (PG) samples were analyzed as described previously [28]. After washing with MQ water, samples were digested with pronase E (100 µg/ml) in a TrisHCl 10 mM pH 7.5 buffer for 1 hour at 60°C to remove Braun's lipoprotein. After heat-inactivation and washing, the samples were treated with muramidase (100 µg/ml) for 16 hours at 37°C, in 50 mM phosphate buffer, pH 4.9. Muramidase digestion was stopped by boiling, coagulated proteins were removed by centrifugation (10 min, 14000 rpm) and the supernatants were reduced with 150 µl 0.5 M sodium borate pH 9.5 and sodium borohydride (10 mg/ml final concentration, 30 min at RT). Finally, samples (100 µl) were adjusted to pH 3.5 with phosphoric acid.

UPLC analyses of muropeptides were performed on an ACQUITY UPLC BEH C18 Column, 130Å, 1.7 µm, 2.1 mm×150 mm (Water, USA) and detected at Abs. 204 nm. Muropeptides were separated using a linear gradient from buffer A (phosphate buffer 50 mM pH 4.35) to buffer B (phosphate buffer 50 mM pH 4.95 methanol 15% (v/v)) in a 20 minutes run.

### Quantification of relative abundances of muropeptides

Identity of the peaks was assigned by comparison of the retention times and profiles to other chromatograms in which mass spectrometry data has been collected. The relative amount of each muropeptide was calculated by comparison of the relative area of the peak compared to the total area of the chromatogram. Representative chromatograms are shown in Fig. S9.

The degree of crosslinking is expressed as the relative amount of peptide bonds that connect two peptide stems ([dimers+trimers/2]). The average length is indirectly proportional to the relative amount of anhydro-muropeptides.

### PG quantifcation

Isolated PG sacculi were hydrolysed for 15 hours with HCl 6M at 100°C, followed by water removal using a centrifugal concentrator (Speed Vac). Completely dried samples were resuspended in water and treated with ninhydrin (250 mg of ninhydrin in 4 ml of phosphoric acid 0.6 M and 6 ml of pure acetic acid) for 5 minutes at 100°C. Absorbance was measured at 434 nm and concentration of muropeptides was calculated by comparison to a mDAP standard curve [29].

### Protein purification

All proteins were overproduced in *E. coli* strain RosettaGami (Invitrogen) as 6×His-tagged constructs from a pET28b+ vector. Overnight cultures of overexpression strains carrying either truncated CsiV_31-266_-His, truncated LpoA_61-653_ or full-length His-PBP1A were diluted into 1 L LB broth, grown until OD_600_ = 0.5. Flasks were then cooled down at 4°C for 30 min, followed by addition of 1 mM IPTG and slow shaking at room temperature for 16 h. Cells were pelleted, washed 1× in PBS and resuspended in Buffer A (20 mM Tris, pH 7.2, 150 mM NaCl, 1 mM DTT+protease inhibitor cocktail (Roche) and stored at −80°C. Following thawing on ice, cells were disrupted by passaging through a French press twice. Salt was then adjusted to 300 mM NaCl and 0.1% triton x-100 (reduced, Sigma) as well as 1% CHAPS (Sigma) and 40 mM Imidazole added. Lysates were then incubated for 1 hour rotating at 4°C, followed by centrifugation for 1 h (25.000 rpm, Beckman Coulter Avanti J26-XP centrifuge, JL-25.50 rotor) at 4°C. Nickel NTA resin (0.5 ml, equilibrated in buffer A) was then added to the supernatant, followed by incubation at 4°C, rotating. The lysate was then allowed to drain from the Ni-resin by flow-through in a filter cartridge and the resin washed (5×10 ml) with Wash buffer (Buffer A adjusted to 500 mM NaCl, 50 mM imidazole and 0.1% triton x-100) and eluted with wash buffer containing an imidazole gradient (60–300 mM). Fractions were subjected to SDS PAGE and Coomassie Brilliant Blue staining and the cleanest fractions pooled. Proteins were quantified using Nanodrop.

### Co-affinity purification

For co-affinity purification, strains carrying chromosomal C-terminal His fusions of the proteins of interest were grown to OD_600_∼0.5 in LB, pelleted, washed twice in PBS and then resuspended in PBS and crosslinked for 30 min with 5 mM DSP (Thermo Scientific). The pellet was then washed 3× in PBS and resuspended in buffer B (20 mM Tris, 300 mM NaCl, 1 mM DTT, 1% Triton X-100, Roche complete protease inhibitor) and cells were lysed by passaging three times through a French Press. Then, 1% CHAPS was added and the lysates stirred 2 h – overnight at 4°C. Lysates were then cleared by centrifugation (25.000 rpm, 1 h) and incubated for 2 h with His-antibody resin (R&D systems) equilibrated in buffer B. The resin was washed 3× with Buffer B adjusted to 500 mM NaCl and protein complexes eluted with Buffer C (100 mM glycine, pH 2.5, 300 mM NaCl, 1 mM DTT, 1% Triton X-100). Proteins were then concentrated ∼10fold using Amicon centrifuge filter units with 10 kDa MW cutoff and subjected to SDS PAGE followed by silver staining. Bands of interest were cut out from the gel and proteins identified via Mass Spectrometry.

### Targeted protein-protein interaction assays

CsiV-his was covalently linked to NHS-activated sepharose resin (Thermo Scientific) using the manufacturer's protocol. Purified LpoA-his and His-PBP1A were added to CsiV-Sepharose equilibrated in 20 mM Tris-HCl (pH 7.2), 150 mM NaCl, 1 mM DTT and 0.1% reduced Triton X-100 and incubated at 4°C rotating for 2 h. The resin was then washed 3× with buffer and proteins eluted by boiling the resin in buffer containing 1% SDS (95°C, 5 min). Proteins were then visualized via Western Blot.

### Bacterial Two Hybrid Analysis (BACTH)

BACTH was conducted using a split adenylate cyclase system as described previously [30].

### PG binding assay

PG binding was assayed as described previously [31]. In short, purified PG sacculi were incubated with purified proteins in pulldown buffer (20 mM Tris/maleate pH 6.8, 50 mM NaCl, 10 mM MgCl2 0.1% Triton X-100) for 30 min on ice, then ultracentrifuged (110.000 rpm). Pellets were washed once in pulldown buffer and proteins in pellets and supernatant fractions visualized by Western Blot.

## Supporting Information

Figure S1 Western blot of csiV-mCherry. Ectopic expression of CsiV-mCherry was induced by addition of 200 µM IPTG for 2 h, followed by lysis and western blotting using anti-mCherry antibody. The two observed bands are consistent with the predicted sizes of CsiV-mCherry +/− signal sequence.
(TIFF)

Figure S2 Co-occurrence patterns of CsiV and VC2168. Data were extracted from the String database (http://string-db.org/) and represent the subset of phyla that contain either CsiV, VC2168 or both.
(TIFF)

Figure S3 Comparison of intestinal colonization in infant mice by wt, *csiV* and *mrcA V. cholerae*. Strains were orally inoculated into suckling mice. Total cfu/mouse in intestinal homogenates was assessed after 24 h.
(TIFF)

Figure S4 Effect of PBP1B depletion on the morphology of Δ*csiV* cells. Cells in which PBP1B's native promoter was replaced by an arabinose-inducible promoter (P_ARA_) were initially grown in the presence of arabinose (ARA+), then resuspended in fresh medium without arabinose (ARA−) and imaged at 1 minute intervals.
(TIFF)

Figure S5 Influence of ShyA expression on the growth of a cefsulodin treated Δ*csiV* Δ*shyA* mutant. Growth curves, based on OD600, for a Δ*csiV* Δ*shyA* derivative which carries a chromosomal *shyA* under IPTG control inserted into a neutral locus (*lacZ*) grown in the presence of 100 µg/ml cefsulodin, IPTG (200 µM), both, or neither. Data are averages of two biological replicates; error bars represent standard deviation.
(TIFF)

Figure S6 ShyA overproduction in Δ*csiV* is not toxic. Exponentially growing, uninduced cells carrying an inducible *shyA* expression construct (p*shyA*) were diluted into fresh medium +/− 500 µM IPTG at 37°C. A representative experiment (of two repetitions with similar results) is shown; data are averages of technical quadruplicates.
(TIFF)

Figure S7 CsiV and LpoA are copurified from *V. cholerae* cell lysates. (A) Lysates of *csiV::csiV-his_6_* cells +/− Dithiobis succinmidyl propionate (DSP) treatment were affinity purified on His-antibody resin. Purified proteins were visualized via silver staining of SDS-PAGE gels, and protein bands of interest were analyzed by mass spectroscopy. (B) Lysates of wt and *lpoA::lpoA-his_6_* cells +/− DSP treatment were affinity purified on His-antibody resin. Purified proteins were identified as in (A).
(TIFF)

Figure S8 The association of LpoA with PG in vivo is independent of CsiV and PBP1A. Soluble (S) and PG-associated (P) proteins were isolated from an *lpoA::lpoA-his_6_* strain and derivatives lacking *csiV*, *mrcA*, or both after DSP crosslinking. Following reversal of crosslinks, the presence of LpoA-His6 in each fraction was monitored by western blotting using an anti-His antibody.
(TIFF)

Figure S9 UPLC chromatograms. Representative chromatograms of muramidase-digested PG samples.
(TIF)

Table S1 Oligos used in this study.
(XLS)

Table S2 Strains and plasmids.
(XLS)
