# Supplementary figures and images for: A Novel Peptidoglycan Binding Protein Crucial for PBP1A-Mediated Cell Wall Biogenesis in Vibrio cholerae
Source: PLoS Genet. 2014 Jun 19;10(6):e1004433. doi: 10.1371/journal.pgen.1004433 (PMC4063736; doi:10.1371/journal.pgen.1004433)

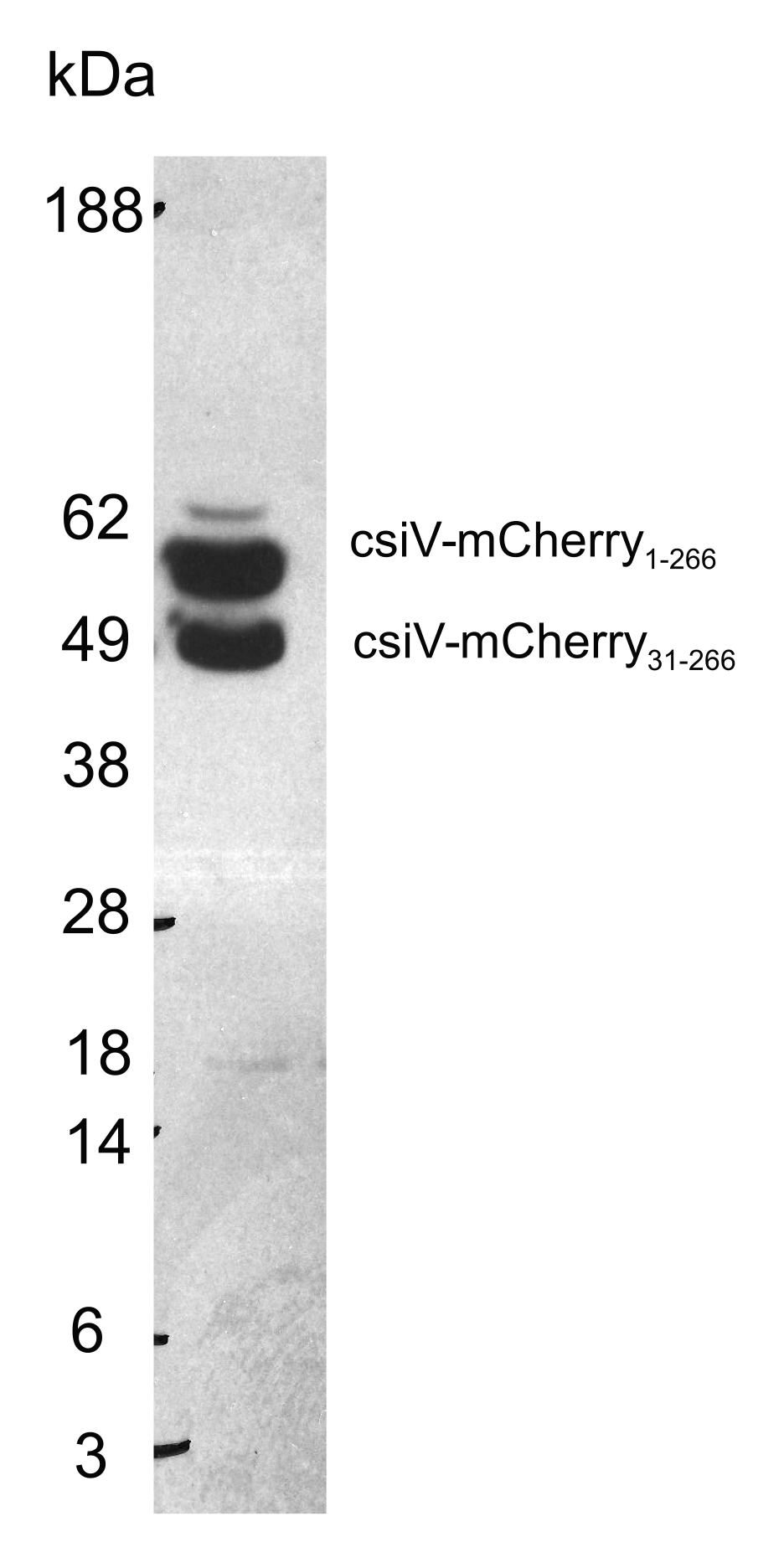

Supplement: Figure S1 — Western blot of csiV-mCherry. Ectopic expression of CsiV-mCherry was induced by addition of 200 µM IPTG for 2 h, followed by lysis and western blotting using anti-mCherry antibody. The two observed bands are consistent with the predicted sizes of CsiV-mCherry +/− signal sequence. (TIFF) [file pgen.1004433.s001.tiff]

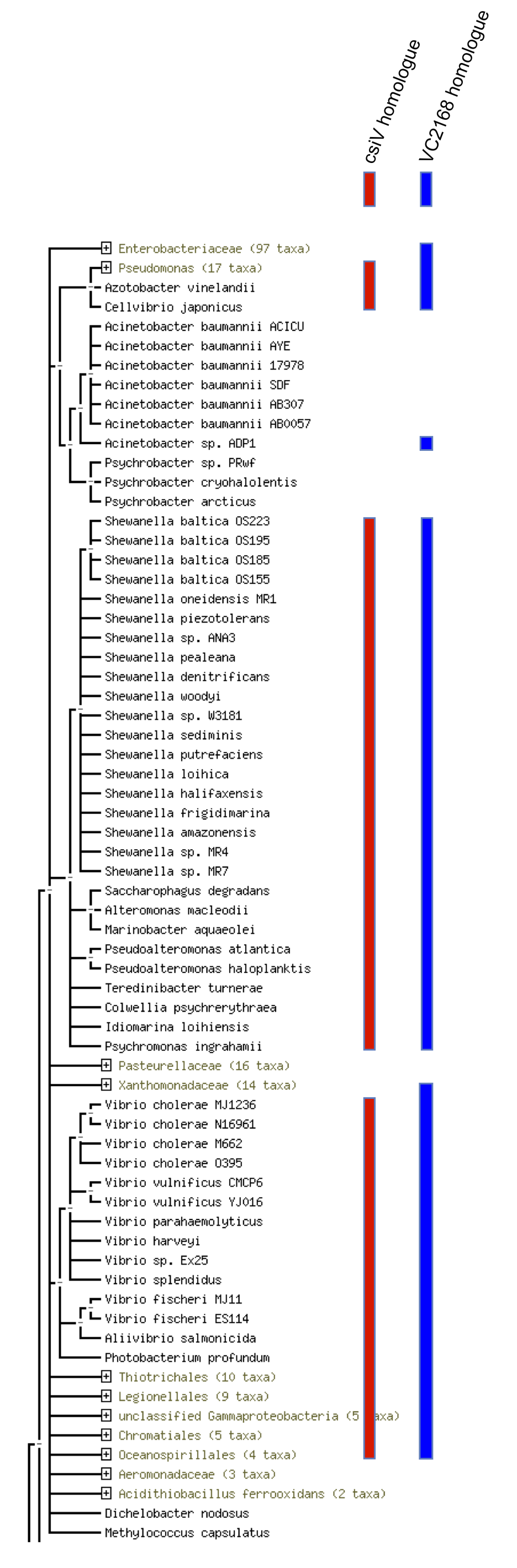

Supplement: Figure S2 — Co-occurrence patterns of CsiV and VC2168. Data were extracted from the String database (http://string-db.org/) and represent the subset of phyla that contain either CsiV, VC2168 or both. (TIFF) [file pgen.1004433.s002.tiff]

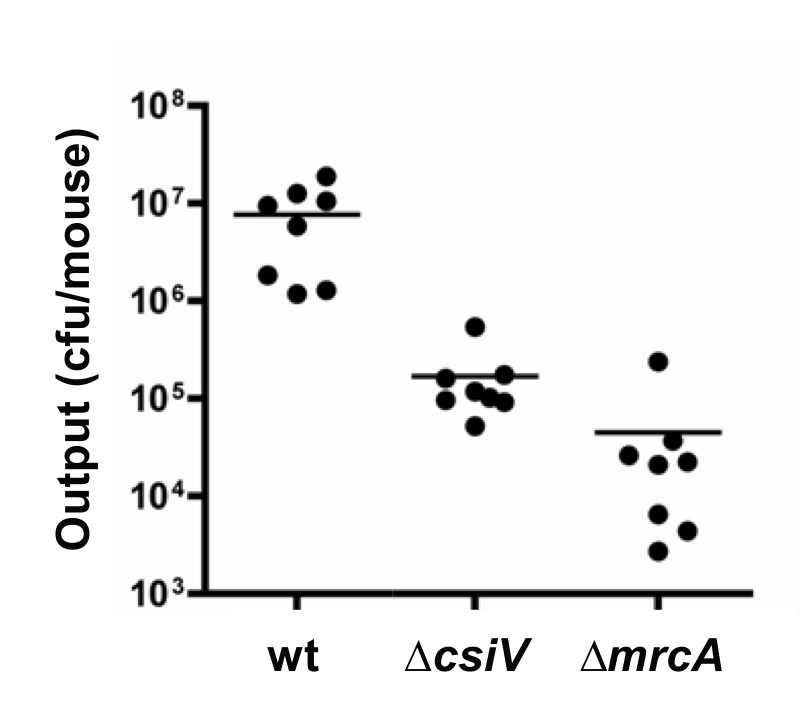

Supplement: Figure S3 — Comparison of intestinal colonization in infant mice by wt, csiV and mrcA V. cholerae. Strains were orally inoculated into suckling mice. Total cfu/mouse in intestinal homogenates was assessed after 24 h. (TIFF) [file pgen.1004433.s003.tiff]

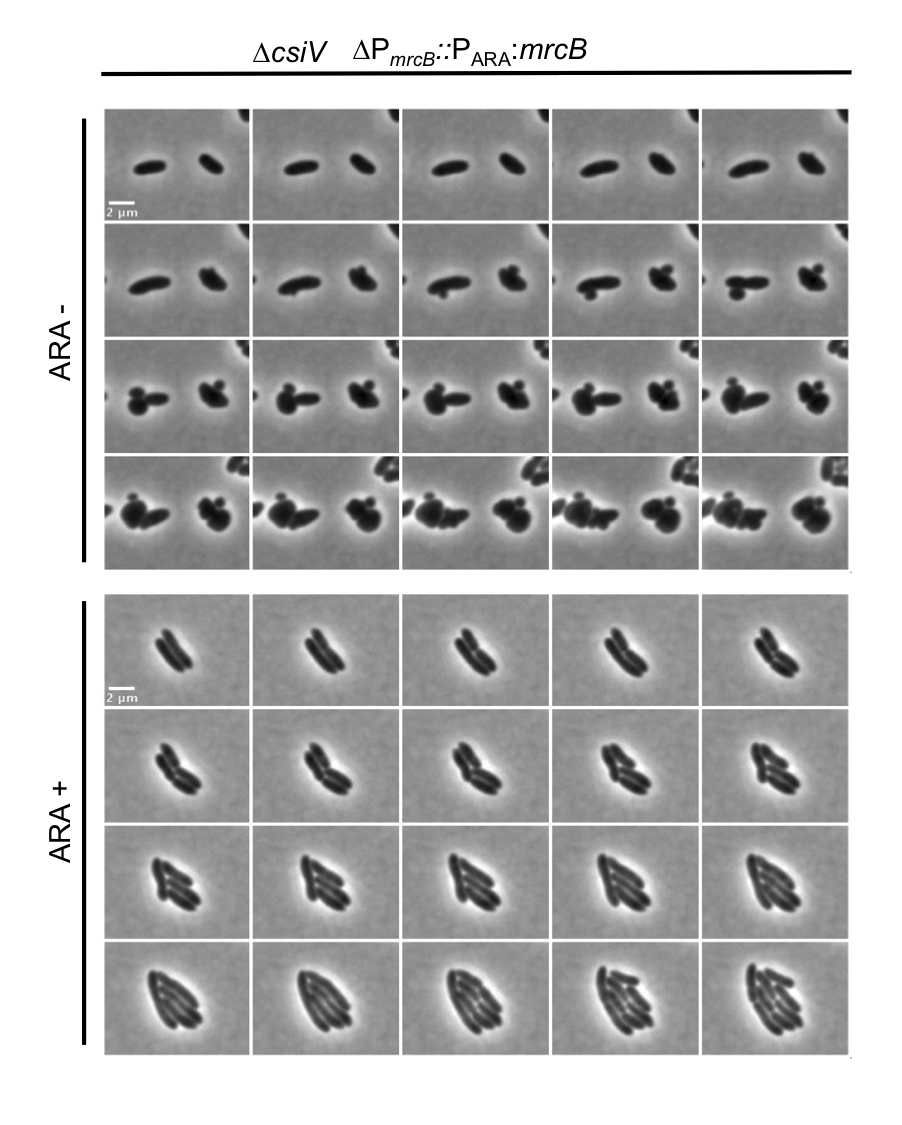

Supplement: Figure S4 — Effect of PBP1B depletion on the morphology of ΔcsiV cells. Cells in which PBP1B's native promoter was replaced by an arabinose-inducible promoter (PARA) were initially grown in the presence of arabinose (ARA+), then resuspended in fresh medium without arabinose (ARA−) and imaged at 1 minute intervals. (TIFF) [file pgen.1004433.s004.tiff]

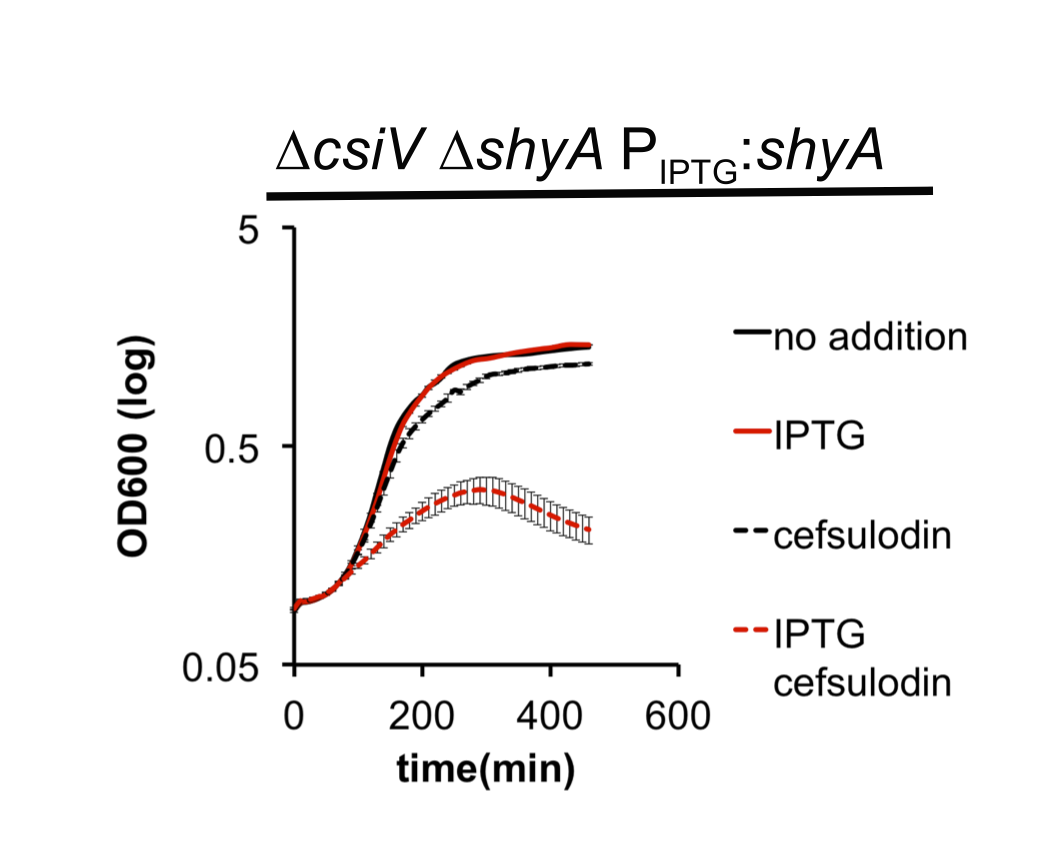

Supplement: Figure S5 — Influence of ShyA expression on the growth of a cefsulodin treated ΔcsiV ΔshyA mutant. Growth curves, based on OD600, for a ΔcsiV ΔshyA derivative which carries a chromosomal shyA under IPTG control inserted into a neutral locus (lacZ) grown in the presence of 100 µg/ml cefsulodin, IPTG (200 µM), both, or neither. Data are averages of two biological replicates; error bars represent standard deviation. (TIFF) [file pgen.1004433.s005.tiff]

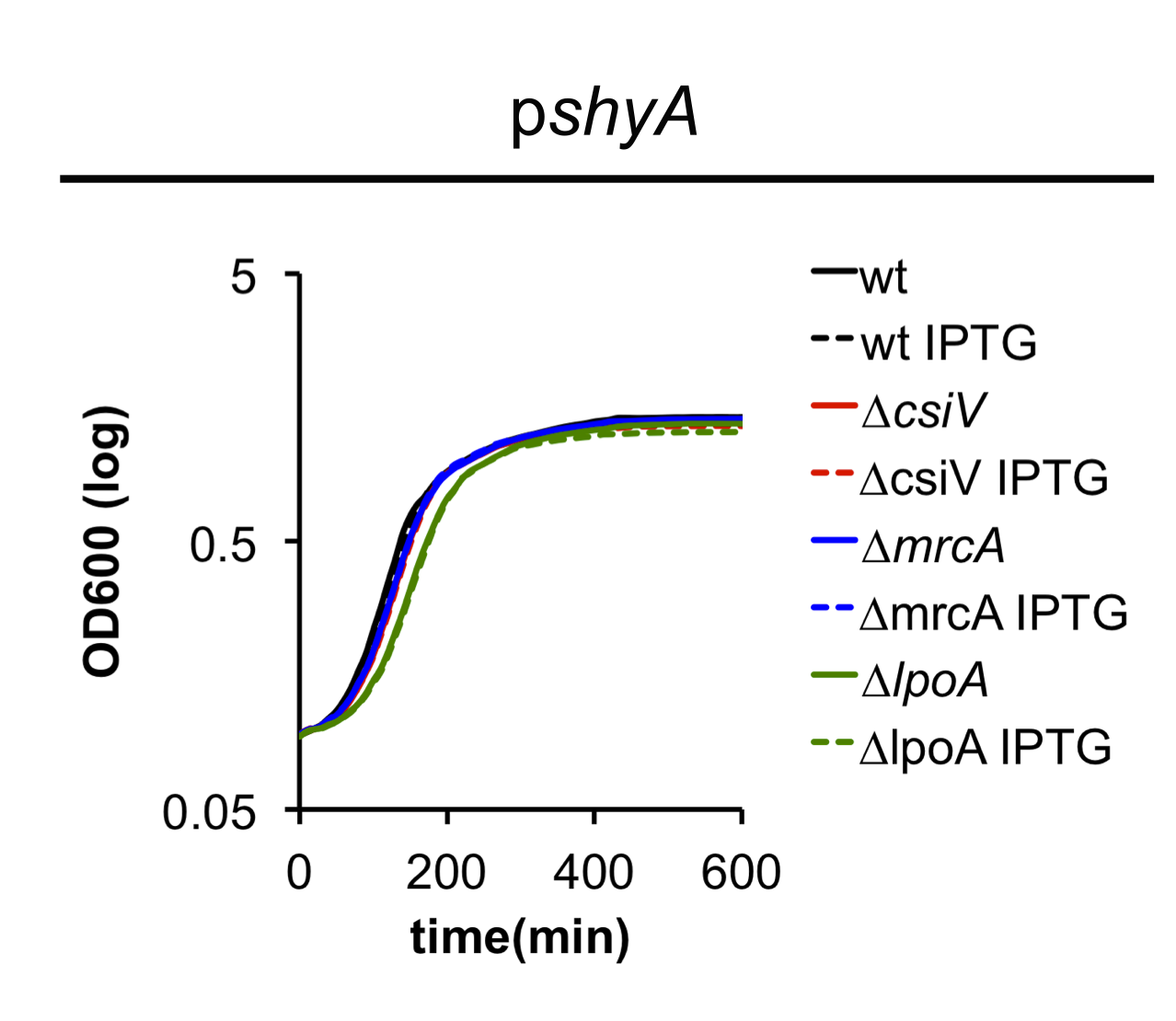

Supplement: Figure S6 — ShyA overproduction in ΔcsiV is not toxic. Exponentially growing, uninduced cells carrying an inducible shyA expression construct (pshyA) were diluted into fresh medium +/− 500 µM IPTG at 37°C. A representative experiment (of two repetitions with similar results) is shown; data are averages of technical quadruplicates. (TIFF) [file pgen.1004433.s006.tiff]

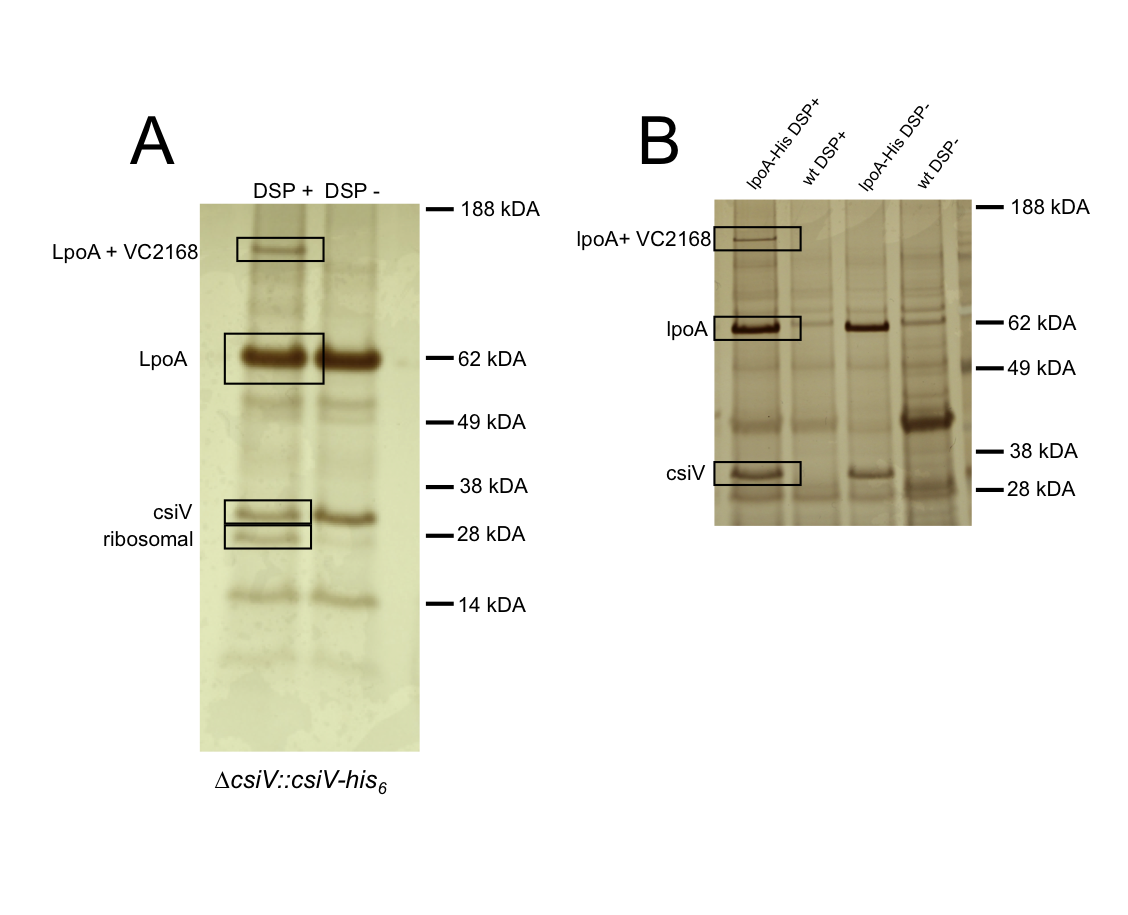

Supplement: Figure S7 — CsiV and LpoA are copurified from V. cholerae cell lysates. (A) Lysates of csiV::csiV-his6 cells +/− Dithiobis succinmidyl propionate (DSP) treatment were affinity purified on His-antibody resin. Purified proteins were visualized via silver staining of SDS-PAGE gels, and protein bands of interest were analyzed by mass spectroscopy. (B) Lysates of wt and lpoA::lpoA-his6 cells +/− DSP treatment were affinity purified on His-antibody resin. Purified proteins were identified as in (A). (TIFF) [file pgen.1004433.s007.tiff]

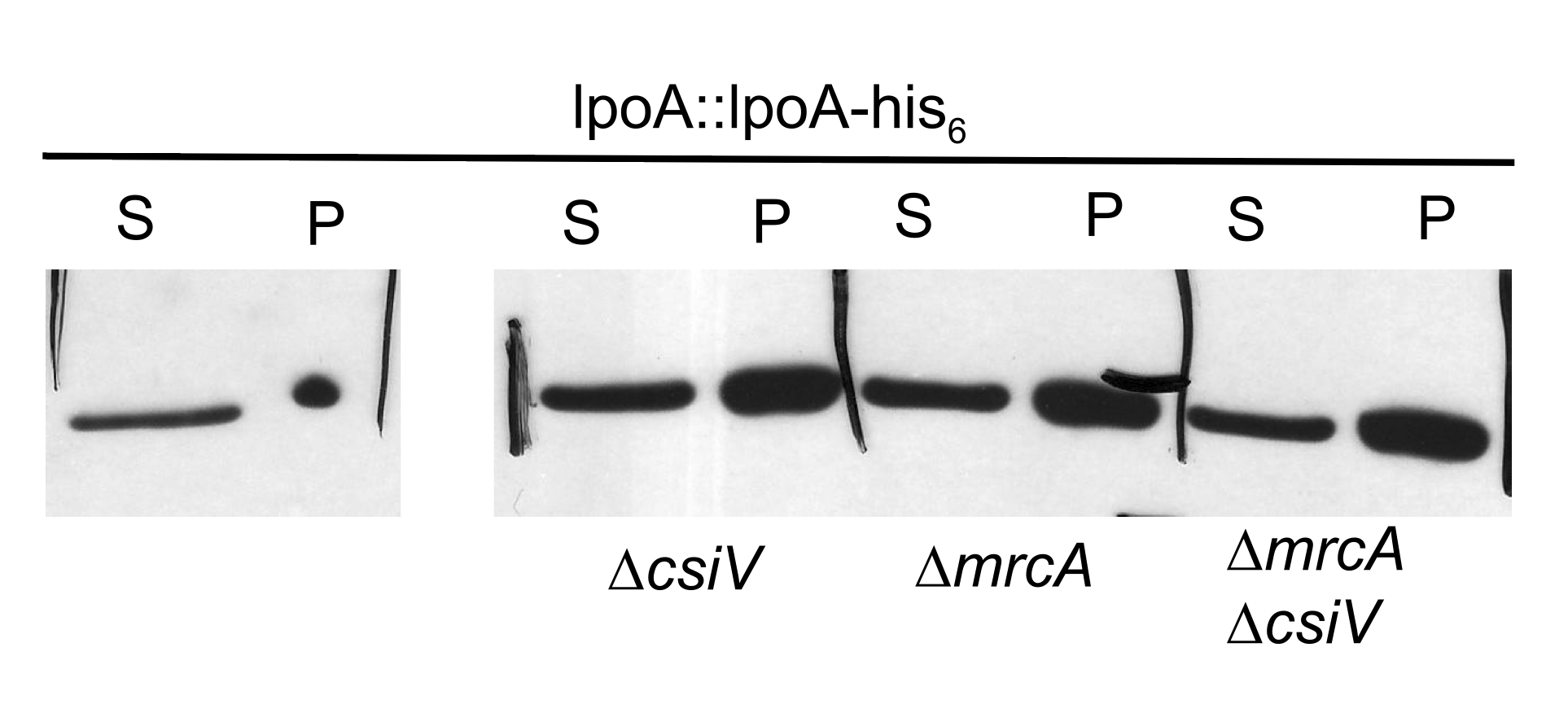

Supplement: Figure S8 — The association of LpoA with PG in vivo is independent of CsiV and PBP1A. Soluble (S) and PG-associated (P) proteins were isolated from an lpoA::lpoA-his6 strain and derivatives lacking csiV, mrcA, or both after DSP crosslinking. Following reversal of crosslinks, the presence of LpoA-His6 in each fraction was monitored by western blotting using an anti-His antibody. (TIFF) [file pgen.1004433.s008.tiff]

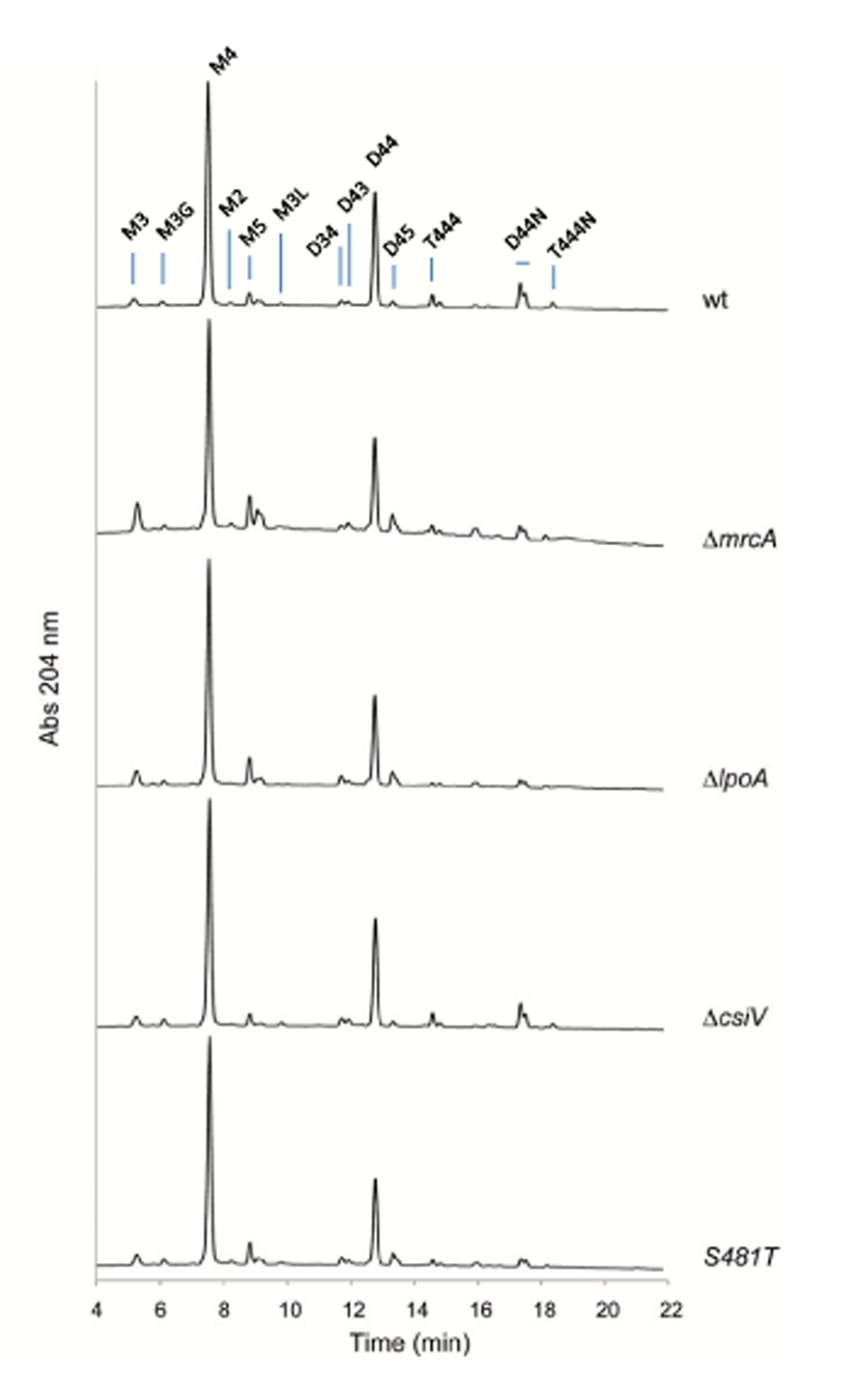

Supplement: Figure S9 — UPLC chromatograms. Representative chromatograms of muramidase-digested PG samples. (TIF) [file pgen.1004433.s009.tif]
